# Observation of ultrafast interfacial Meitner-Auger energy transfer in a Van der Waals heterostructure

**DOI:** 10.1038/s41467-023-40815-8

**Published:** 2023-08-19

**Authors:** Shuo Dong, Samuel Beaulieu, Malte Selig, Philipp Rosenzweig, Dominik Christiansen, Tommaso Pincelli, Maciej Dendzik, Jonas D. Ziegler, Julian Maklar, R. Patrick Xian, Alexander Neef, Avaise Mohammed, Armin Schulz, Mona Stadler, Michael Jetter, Peter Michler, Takashi Taniguchi, Kenji Watanabe, Hidenori Takagi, Ulrich Starke, Alexey Chernikov, Martin Wolf, Hiro Nakamura, Andreas Knorr, Laurenz Rettig, Ralph Ernstorfer

**Affiliations:** 1 Fritz-Haber-Institut der Max-Planck-Gesellschaft, Faradayweg 4-6, 14195 Berlin, Germany; 2 Beijing National Laboratory for Condensed Matter Physics, Institute of Physics, Chinese Academy of Sciences, Beijing, 100190 China; 3 Université de Bordeaux - CNRS - CEA, CELIA, UMR5107, F33405 Talence, France; 4 Nichtlineare Optik und Quantenelektronik, Institut für Theoretische Physik, Technische Universität Berlin, 10623 Berlin, Germany; 5 Max Planck Institute for Solid State Research, 70569 Stuttgart, Germany; 6 Department of Applied Physics, KTH Royal Institute of Technology, Hannes Alfvéns väg 12, 114 19 Stockholm, Sweden; 7 Institute of Applied Physics and Würzburg-Dresden Cluster of Excellence ct.qmat, Technische Universität Dresden, 01062 Dresden, Germany; 8 Photonics Laboratory, ETH Zürich, 8093 Zürich, Switzerland; 9 Department of Statistical Sciences, University of Toronto, 700 University Avenue, Toronto, ON M5G 1Z5 Canada; 10 Institute of Semiconductor Optics and Functional Interfaces, Research Center SCoPE and IQST, University of Stuttgart, 70569 Stuttgart, Germany; 11 International Center for Materials Nanoarchitectonics, National Institute for Materials Science, 1-1 Namiki, Tsukuba, 305-0044 Japan; 12 Research Center for Electronic and Optical Materials, National Institute for Materials Science, 1-1 Namiki, Tsukuba, 305-0044 Japan; 13 Department of Physics, University of Tokyo, 113-0033 Tokyo, Japan; 14 Institute for Functional Matter and Quantum Technologies, University of Stuttgart, 70569 Stuttgart, Germany; 15 Department of Physics, University of Arkansas, Fayetteville, AR 72701 USA; 16 Institut für Optik und Atomare Physik, Technische Universität Berlin, 10623 Berlin, Germany

**Keywords:** Surfaces, interfaces and thin films, Two-dimensional materials

## Abstract

Atomically thin layered van der Waals heterostructures feature exotic and emergent optoelectronic properties. With growing interest in these novel quantum materials, the microscopic understanding of fundamental interfacial coupling mechanisms is of capital importance. Here, using multidimensional photoemission spectroscopy, we provide a layer- and momentum-resolved view on ultrafast interlayer electron and energy transfer in a monolayer-WSe_2_/graphene heterostructure. Depending on the nature of the optically prepared state, we find the different dominating transfer mechanisms: while electron injection from graphene to WSe_2_ is observed after photoexcitation of quasi-free hot carriers in the graphene layer, we establish an interfacial Meitner-Auger energy transfer process following the excitation of excitons in WSe_2_. By analysing the time-energy-momentum distributions of excited-state carriers with a rate-equation model, we distinguish these two types of interfacial dynamics and identify the ultrafast conversion of excitons in WSe_2_ to valence band transitions in graphene. Microscopic calculations find interfacial dipole-monopole coupling underlying the Meitner-Auger energy transfer to dominate over conventional Förster- and Dexter-type interactions, in agreement with the experimental observations. The energy transfer mechanism revealed here might enable new hot-carrier-based device concepts with van der Waals heterostructures.

## Abstract (web summary)

Here, the authors investigate the interfacial charge/energy transfer dynamics in a WSe_2_/graphene heterostructure. They unveil an energy transfer mechanism from WSe_2_ to graphene mediated by an interfacial Meitner-Auger process, resulting in a transient hole distribution in the Dirac cone at energies larger than the photon energy of the optical excitation.

## Introduction

The unique physical properties of atomically thin two-dimensional (2D) materials^1,2^ and constantly improving fabrication methods^3,4^ have led to a great interest in novel quantum materials based on van der Waals (vdW) heterostructures^5^. By stacking 2D materials, vdW heterostructures inherit the properties from individual constituents, and exotic physical phenomena may emerge due to the interfacial interaction^5–7^. An emblematic example is the emergence of superconductivity in twisted bilayer graphene when stacked at the so-called “magic angle”^8^. As another example, interlayer excitons, which are spatially separated yet Coulomb-bound electron-hole pairs in semiconducting transition metal dichalcogenide (TMDC) heterostructures allow exceptional control of optoelectronic properties^9–11^. Out of the vdW heterostructure library, a basic optoelectronic building block is a monolayer (ML) semiconducting TMDC in contact with graphene^12^. This hybrid structure represents a model system as it combines the strong light-matter coupling of TMDCs and the high mobility of massless Dirac carriers of graphene^13^. The gapless electronic structure of graphene allows for harvesting low-energy photons, extending the spectral range covered by conventional photodetectors to the near-infrared wavelength, which is highly beneficial for photovoltaic applications^14^.

Optoelectronic functionality in vdW heterostructures arises from careful design and control of optical transitions and interfacial transfer processes. Particularly, interfacial charge (ICT) and energy transfer (IET) are key processes that have triggered extensive experimental and theoretical efforts^15–20^ Using time-resolved optical spectroscopies, a strong reduction of the exciton lifetime^21^ and optically active charge-transfer excitations of TMDC/graphene heterostructures have been observed^22,23^, suggesting strong interlayer coupling and the underlying mechanisms have been discussed^24–26^. Moreover, the efficiency of IET processes like Förster-type coupling (based on electronic dipole-dipole interaction) has recently been investigated theoretically, pointing out the importance of energy-momentum conservation between participating quasiparticles^15^. These studies provide our current understanding of the mechanisms of interfacial interactions. However, it is still challenging to clearly distinguish and unravel the involved interlayer charge and energy transfer in vdW heterostructures based on optical spectroscopies, primarily transient absorption/reflection and terahertz spectroscopy, which are inherently sensitive to the selective spectral range and limited momentum accessible. Therefore, a momentum-resolved probe is required to monitor the dynamics directly and achieve a complete picture of interfacial charge and energy transfer processes, including those involving momentum-forbidden dark states.

**Fig. 1:**
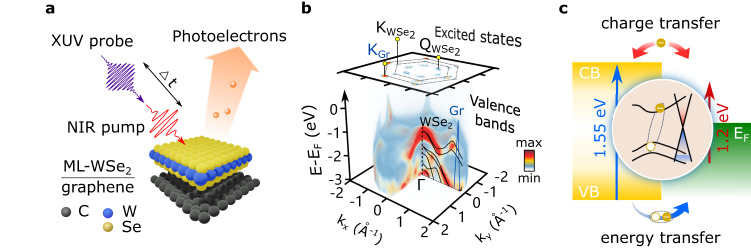
Time- and angle-resolved photoemission measurement of interlayer charge and energy transfer in a ML-WSe_2_/graphene heterostructure. **a** Following the near-infrared pump, electrons are photoionized by the delayed XUV-probe pulses and collected by a three-dimensional (3D) (*E*_*kin*_, *k*_*x*_, *k*_*y*_) detector as a function of pump-probe delay Δ*t*. **b** The 3D snapshot of the 4D data, *I*(*E*_*kin*_, *k*_*x*_, *k*_*y*_, Δ*t* = 0 fs) presents the valence band structures from the Γ point to the Brillouin zone boundary of WSe_2_, as well as the linearly dispersing graphene bands. The excited state population can be clearly mapped at the ${{{{{{{{\rm{K}}}}}}}}}_{{{{{{{{{\rm{WSe}}}}}}}}}_{{{{{{{{\rm{2}}}}}}}}}}$ and ${{{{{{{{\rm{Q}}}}}}}}}_{{{{{{{{{\rm{WSe}}}}}}}}}_{{{{{{{{\rm{2}}}}}}}}}}$ valleys, and the π^*^ band of graphene (K_Gr_). **c** By changing the pump wavelength, we can selectively prepare different initial excited states: quasi-free carriers in graphene with below-bandgap excitation (red arrow) or excitons in WSe_2_ using excitation on the excitonic resonance (blue arrow).

Here, we use time- and angle-resolved photoemission spectroscopy (trARPES) to investigate ultrafast interlayer carrier interactions in an epitaxially grown ML-WSe_2_/graphene heterostructure. Our trARPES setup combines a high-repetition-rate (500 kHz) femtosecond extreme ultraviolet (XUV) source^27^ coupled to a time-of-flight momentum microscope^28^ (see Methods). It allows the measurement of the four-dimensional (4D) photoemission intensity *I*(*E*_kin_, *k*_*x*_, *k*_*y*_, Δ*t*), where *E*_*kin*_ is the outgoing photoelectron kinetic energy, *k*_*x*_,*k*_*y*_ are the in-plane momenta and Δ*t* is the pump-probe delay, as shown in Fig. 1a, b. The probe photon energy of 21.7 eV allows accessing the entire Brillouin zone of the heterostructure and the variable pump wavelength allows us to photoexcite the heterostructure in a state-resolved manner. In the following, we present a time-, energy-, and momentum-resolved study on the excited-state dynamics in the heterostructure with two different pump photon energies: below the optical bandgap of WSe_2_ (1.2 eV) and in resonance with its first excitonic transition (1.55 eV).

## Results

### Interlayer quasi-free carrier transfer

**Fig. 2:**
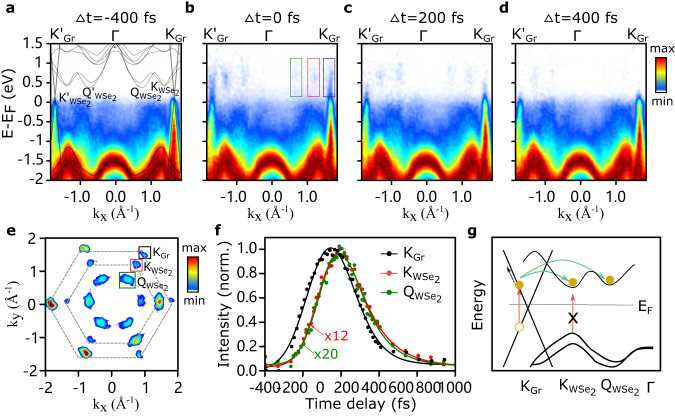
Layer- and valley-resolved ultrafast dynamics upon below-bandgap pumping. **a**–**d** Energy-momentum cuts of the photoemission signal along the ${{{{{{{{\rm{{K}}}}}}}^{{\prime} }}}}_{{{{{{{{\rm{Gr}}}}}}}}}$- ${{{{{{{{\rm{{K}}}}}}}^{{\prime} }}}}_{{{{{{{{{\rm{WSe}}}}}}}}}_{2}}$-Γ-${{{{{{{{\rm{K}}}}}}}}}_{{{{{{{{{\rm{WSe}}}}}}}}}_{2}}$-K_Gr_ high symmetry direction, at selected pump-probe time delays. **a** The 2D spectrum at negative time delay reveals the equilibrium band structure of ML-WSe_2_ as well as the linearly dispersing *π* band of graphene. The gray lines represent the DFT-calculated band structures (details in methods). Snapshots of the energy-momentum cuts at time delays of **b** Δ*t* = 0 fs, **c** Δ*t* = 200 fs, and **d** Δ*t* = 400 fs, respectively. **e** Momentum map of the excited states (energy integrated for *E* > *E*_F_ and time integrated for the first 400 fs), showing the K_Gr_ points of graphene (black box) as well as the ${{{{{{{{\rm{K}}}}}}}}}_{{{{{{{{{\rm{WSe}}}}}}}}}_{{{{{{{{\rm{2}}}}}}}}}}$ and ${{{{{{{{\rm{Q}}}}}}}}}_{{{{{{{{{\rm{WSe}}}}}}}}}_{{{{{{{{\rm{2}}}}}}}}}}$ valleys (red and green boxes, respectively). The dashed gray lines represent the hexagonal Brillouin zones of both layers. **f** Normalized population dynamics within the three ROIs defined in **e**: ${{{{{{{{\rm{K}}}}}}}}}_{{{{{{{{{\rm{WSe}}}}}}}}}_{{{{{{{{\rm{2}}}}}}}}}}$ (red) and ${{{{{{{{\rm{Q}}}}}}}}}_{{{{{{{{{\rm{WSe}}}}}}}}}_{{{{{{{{\rm{2}}}}}}}}}}$ (green) are populated with a ~50 fs delay with respect to K_Gr_ (black). **g** Schematic of the early-time carrier dynamics upon below-bandgap excitation: photo-generated hot carriers within the graphene layer are transferred to the conduction bands of WSe_2_ via hot electron injection after the thermalization.

First, we photoexcite the heterostructure with the pump photon energy centered at *ℏω*_pump_=1.2 eV (pump pulse duration 200 fs FWHM), well below the optical bandgap of WSe_2_^29^. The NIR-pump/XUV-probe experiments were performed with a pump fluence of *F* = 5.3 mJ/cm^2^ and at room temperature. Figure 2a–d shows energy-resolved photoemission signals along the ${{{{{{{\rm{{K}}}}}}}^{{\prime} }}}-{{{{{{{\rm{K}}}}}}}}$ cut of the Brillouin zone, at selected time delays. The band mappings are contrast-enhanced using a multidimensional extension of the contrast limited adaptive histogram equalization (MCLAHE)^30,31^ for better visualization of the band structure. The momentum distributions above *E*_*F*_ within the first 400 fs reveal that the excited states are localized in three different types of valleys: the Dirac cones of graphene at its K points (K_Gr_) and the K and Q valleys of WSe_2_ (${{{{{{{\rm{{K}}}}}}}_{WS{e}_{2}},{{{{{{{{\rm{Q}}}}}}}}}_{{{{{{{{{\rm{WSe}}}}}}}}}_{{{{{{{{\rm{2}}}}}}}}}}}}$), as shown in Fig. 2e. The ${{{{{{{{\rm{Q}}}}}}}}}_{{{{{{{{{\rm{WSe}}}}}}}}}_{{{{{{{{\rm{2}}}}}}}}}}$ valley localizes between the ${{{{{{{{\rm{K}}}}}}}}}_{{{{{{{{{\rm{WSe}}}}}}}}}_{{{{{{{{\rm{2}}}}}}}}}}$ valley and the Γ point. By performing energy-momentum integration in selected regions of interest (ROIs), we extracted excited-state dynamics within these three valleys (Fig. 2f). Upon arrival of the pump pulses, the excited-state population rapidly builds up at K_Gr_ (black curve) and decays with a time scale of ~200 fs. Strikingly, the conduction band minima (CBMs) at ${{{{{{{{\rm{K}}}}}}}}}_{{{{{{{{{\rm{WSe}}}}}}}}}_{{{{{{{{\rm{2}}}}}}}}}}$ (red curve) and ${{{{{{{{\rm{Q}}}}}}}}}_{{{{{{{{{\rm{WSe}}}}}}}}}_{{{{{{{{\rm{2}}}}}}}}}}$ valleys (green curve) are also being populated, however, with a delay of Δ*t* = 51 ± 9 fs (see SI) compared to the rise of hot-carrier population in graphene. Since below-bandgap pump photon energy does not allow the direct photoexcitation of WSe_2_, the delayed electron populations in the conduction bands arise through charge transfer from graphene to WSe_2_. Two/multiple photon excitation can safely be ruled out (details see SI). The excited-state population of the ${{{{{{{{\rm{Q}}}}}}}}}_{{{{{{{{{\rm{WSe}}}}}}}}}_{{{{{{{{\rm{2}}}}}}}}}}$ valleys (Fig. 2f) could be raised via ICT from the graphene layer and the intervalley scattering from the ${{{{{{{{\rm{K}}}}}}}}}_{{{{{{{{{\rm{WSe}}}}}}}}}_{{{{{{{{\rm{2}}}}}}}}}}$ valleys^32,33^.

These observations support the following picture of the underlying processes with a below-bandgap excitation: light is absorbed by graphene and populates unoccupied states at ${E}_{Gr}^{el}={E}_{D}+\hslash {\omega }_{{{{{\rm{pump}}}}}}/2$, leaving holes at ${E}_{Gr}^{h}={E}_{D}-\hslash {\omega }_{{{{{\rm{pump}}}}}}/2$ (Dirac energy *E*_*D*_ > 0 for a p-doped system or *E*_*D*_ < 0 for an n-doped system). The energy position of the Dirac point in our heterostructure is estimated to be ~ −0.1eV below the Fermi level, obtained from the conical crossing^34,35^ (see SI). The photoexcited carriers quickly reach a quasi-thermalized states in ~10 fs^36^ and could further increase their energy via intraband electron-electron scattering and interband Auger recombination in few tens of femtoseconds^37,38^. Once electrons gained a sufficient amount of energy to overcome the energy barrier, they scatter to WSe_2_ via a phonon-assisted tunneling process, filling the single-particle CBMs at ${{{{{{{{\rm{K}}}}}}}}}_{{{{{{{{{\rm{WSe}}}}}}}}}_{{{{{{{{\rm{2}}}}}}}}}}$ and ${{{{{{{{\rm{Q}}}}}}}}}_{{{{{{{{{\rm{WSe}}}}}}}}}_{{{{{{{{\rm{2}}}}}}}}}}$. This ICT mechanism is called interlayer hot-carrier injection, and is schematically illustrated in Fig. 2g. The excited electrons in WSe_2_ may subsequently scatter back to graphene and relax down towards the Fermi energy (*E*_*F*_). Based on the observed carrier dynamics, we performed microscopic calculations of the phonon-assisted interlayer tunneling process, allowing us to estimate the electronic wavefunction overlap between the involved conduction bands of WSe_2_ and graphene to be ~4% (see SI for details).

### Interlayer energy transfer

**Fig. 3:**
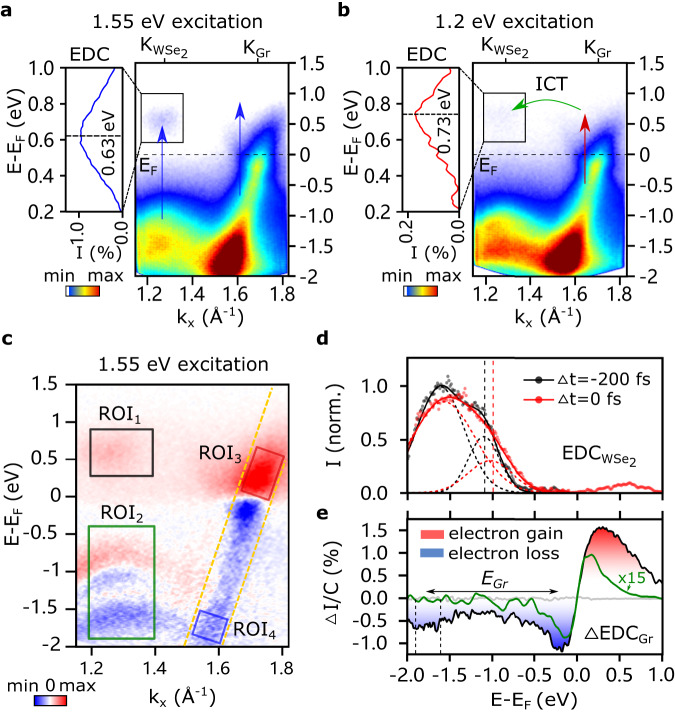
Photoemission signatures of exciton formation and interfacial interactions. **a** With near-resonant A-exciton pump (1.55 eV), carriers within both the WSe_2_ and the graphene layer are photoexcited (time integration of 100 fs). The energy of the excited-states carriers at ${{{{{{{{\rm{K}}}}}}}}}_{{{{{{{{{\rm{WSe}}}}}}}}}_{{{{{{{{\rm{2}}}}}}}}}}$ is 0.63 eV, shown in the EDC (left panel figure). **b** With below-bandgap excitation (1.2 eV), the local CBM of ${{{{{{{{\rm{K}}}}}}}}}_{{{{{{{{{\rm{WSe}}}}}}}}}_{{{{{{{{\rm{2}}}}}}}}}}$ is filled with ICT-induced electrons and centered at 0.73 eV. **c** Differential energy-momentum cut with 1.55 eV pump at time zero, obtained by subtracting the negative time delay spectrum. **d** The normalized EDC of ${{{{{{{{\rm{K}}}}}}}}}_{{{{{{{{{\rm{WSe}}}}}}}}}_{{{{{{{{\rm{2}}}}}}}}}}$ (momentum integration of 0.2 Å^−1^) at Δ*t* = −200 fs (black) and Δ*t* = 0 fs (red). The VBs are fitted with two Gaussian functions (dashed curves) and the positions of VB1 are indicated by the dash lines. **e** The momentum-integrated spectrum of graphene Dirac bands (between the dashed yellow lines in **c**) shows the electron gain (positive, red area) and loss (negative, blue area) following photoexcitation. The intensity is normalized by the total electron count C obtained from negative time delay spectrum. Apart from the carriers accumulation near the *E*_*F*_, the hole population forms another prominent peak around *E*−*E*_*F*_ = −1.8 eV, indicated between the dash lines. The EDC of graphene with 1.2 eV pump (green) is also shown as a comparison.

Next, we select a pump photon energy of *ℏω*_pump_ = 1.55 eV (pump pulse duration: 35 fs FWHM, pump fluence: *F* = 1.7 mJ/cm^2^), near-resonant to the A-excitonic transition of WSe_2_. In this case, the pump photon energy allows both the WSe_2_ and the graphene layer to be simultaneously photoexcited. One striking observation is that the energy distribution of excited carriers at the ${{{{{{{{\rm{K}}}}}}}}}_{{{{{{{{{\rm{WSe}}}}}}}}}_{{{{{{{{\rm{2}}}}}}}}}}$ valleys is centered at 0.63 eV (Fig. 3a), ~100 meV lower than with below-bandgap excitation (Fig. 3b), as apparent from the energy distribution curves (EDCs) (first 100 fs). As discussed above, with 1.2 eV excitation, the ${{{{{{{{\rm{K}}}}}}}}}_{{{{{{{{{\rm{WSe}}}}}}}}}_{{{{{{{{\rm{2}}}}}}}}}}$ valleys are filled with quasi-free electrons that have tunneled from the graphene layer. Therefore, this ~100 meV energy difference is a direct photoemission signature of exciton formation, when near-resonantly pumping using 1.55 eV photons^39^: the bound electron-hole (el-h) pair reduces the quasi-free particle bandgap by the exciton binding energy. In addition to this excitonic feature, we also observe a transient shift of WSe_2_ valence bands. In Fig. 3d, EDCs at ${{{{{{{{\rm{K}}}}}}}}}_{{{{{{{{{\rm{WSe}}}}}}}}}_{{{{{{{{\rm{2}}}}}}}}}}$ are shown at Δ*t* = 0 fs (red) and Δ*t* = − 200 fs (black), in which the top two valence bands, VB1 and VB2, are fitted using Gaussian lineshape functions (see SI). The peak position of VB1 shifts towards the conduction band within the first 100 fs, transiently shrinking the electronic bandgap. This is due to the arrival of ICT-induced charge carriers from the graphene layer. With near-resonantly pumping the A-exciton, the occurrence of ICT and injection of quasi-free carriers from graphene to WSe_2_ is expected, similar to the case of below-bandgap excitation. This could lead to dynamical screening effect and the observed bandgap renormalization, as reported in highly-excited or doped ML TMDC materials^40–44^. As the magnitude of such a transient bandgap renormalization has been shown to scale with the excited charge carrier density^42,45^, we utilize the VB shift in the following as a measure of the ICT transferred carriers dynamics from graphene layer.

**Fig. 4:**
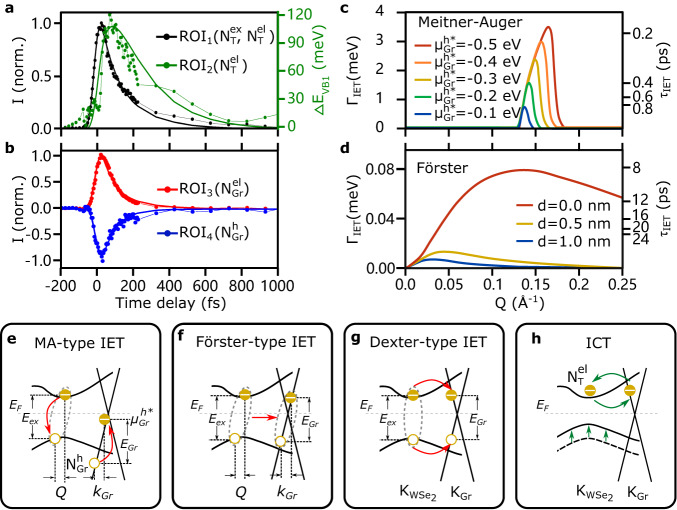
Interlayer charge and energy transfer upon near-resonant A-exciton excitation. **a** By integrating the ROI_1_ in Fig. 3c, the time trace of the normalized photoemission intensity of excited-state carriers at the CBM of WSe_2_ (black) contains the dynamics of excitons (${N}_{T}^{ex}$) and ICT-induced quasi-free carriers (${N}_{T}^{el}$). The energy shift of VB1 (green) mainly reflects the dynamic of ${N}_{T}^{el}$, which are extracted from time-dependent EDCs in ROI_2_. **b** The time traces of hot electrons (red) and hot holes in the deep VB (blue) in graphene are extracted from the ROI_3_ and ROI_4_ in Fig. 3c, respectively. The time traces in **a**, **b** are fitted globally based on a rate-equation model (see text). **c** Calculated Meitner-Auger mediated IET transfer rate as a function of COM momentum **Q** with different photo-induced hole vacancy at $E={\mu }_{Gr}^{h*}$. **d** Calculated Förster coupling rate as a function of **Q** with varied interlayer distance of *d*. Sketch of the underlying carrier dynamics: **e** Meitner-Auger IET with creation of intraband electron-hole pairs in graphene by absorbing the exciton energy. **f** Förster-type energy transfer with the generation of interband electron-hole pairs in graphene. **g** Dexter-type energy transfer with electrons and holes injection to graphene simultaneously. **h** ICT-induced hot electron injection into WSe_2_ and transient energy shift of its valence band.

In addition to the excited-state dynamics in WSe_2_, important insight can be drawn from the energy-momentum distribution of hot carriers in graphene. As shown in the early-time 2D differential spectrum Δ*I*(*E*, *k*, Δ*t* = 0 fs) (Fig. 3c), obtained by subtracting the spectrum at the negative time, hot carriers distribute in a broad energy range. The momentum-integrated spectrum along the linearly dispersing band in Fig. 3e clearly features the energy distribution of net electron gain (positive; red area) and loss (negative; blue area) following near-resonant photoexcitation. Remarkably, besides the modification of the distribution function near the Fermi level, we notice a strong negative peak at *E*−*E*_*F*_ = −1.8 eV. As noted earlier, for direct photoexcitation in graphene the photoexcited carriers are expected to be spread ±0.77eV (*ℏω*_pump_/2) around the Dirac point and quickly relax back to the Fermi level. Thus, this simple excitation mechanism cannot explain this peculiar feature in the valence band spectrum. The electron-electron scattering and Auger recombination could lead to a transient broadening of the momentum-space carrier distribution, but without any preferential energy localization^38,46,47^. hole transfer can also be ruled out, as the top valence band of WSe_2_ lies at *E*−*E*_*F*_ = −1.0 eV. It would require a multi-phonon absorption to populate the hole-states localized deeply in the valence band, taking the typical phonon energy of ~0.17 eV in graphene^48^, a process of very low probability. However, the energy difference of deep-lying valence holes (*E*−*E*_*F*_ = −1.8eV) and states near *E*_*F*_ (*E*−*E*_*F*_ = −0.2 eV) in graphene well matches the energy of the A-exciton in WSe_2_ (*E*_*ex*_ ~ 1.6eV). Combined with the fast depletion of exciton population shown in Fig. 4a (black curve) extracted from the excited state of WSe_2_ (ROI_1_ in Fig. 3c), this brings about the following scenario for the excitation of these carriers: annihilation of excitons in WSe_2_ drives the intraband excitation of deep-lying valence electrons in graphene into empty hole states below the Dirac point. In more detail, this exciton energy transfer process, which we term Meitner-Auger energy transfer^49,50^, considers recombination of excitons in WSe_2_ with center-of-mass (COM) momentum **Q** and exciton energy *E*_*ex*_. The photoexcitation prepares the required hot hole vacancy below *E*_*F*_ in graphene, thus enabling the intraband excitation. The photo-generated hole density plays an important role in the MA-type IET process (see the discussion of pump fluence dependence in SI). Besides the observation of the deep-lying hot holes, we also identify a substantial suppression of hole-like spectral weight (Meitner-Auger type IET-induced hot electrons) below the Fermi level with near-resonant excitation, supporting the occurrence of intraband transition in the graphene layer (details see SI, section Meitner-Auger type IET-induced hot electrons near the Fermi level). The momentum of the valence electron-hole pair *k*_*Gr*_ is determined by the Fermi velocity of the graphene bands and the transition energy *E*_*Gr*_. This required momentum is provided by the optically pumped excitons which gain finite COM momenta during the population formation process via phonon-mediated dephasing and intravalley thermalization^51–54^ (see the discussion in SI). The highly efficient IET of the excitons and intraband electron-hole pairs is thus possible under the conservation of energy and momentum, i.e., *E*_*ex*_ = *E*_*Gr*_ and **Q** = *k*_*Gr*_. In a similar trARPES study of a ML WS_2_/graphene heterostructure, dominating interfacial charge transfer has been observed^17^. Compared with our study, the different charge transfer rates could be raised from the different band structure alignment near the interface and the density of defect sites^26^. While the additional exciton energy transfer was not excluded, its relative efficiency might be reduced due to the larger COM momentum required at the larger A-exciton energy of WS_2_ and the energy level alignment of these specific samples.

In order to gain information on the time scales of the energy and charge transfer processes, next we analyze the dynamics of excited-state populations extracted from the ROIs shown in Fig. 3c, including the excited-state carriers in WSe_2_ (ROI_1_), VB1 shifting (ROI_2_), hot electrons in graphene (ROI_3_) and IET-driven deep valence band holes (ROI_4_). The time trace of hot carriers in the CBM of WSe_2_ (black curve in Fig. 4a) contains two types of quasiparticles dynamics: the photo-generated excitons ${N}_{T}^{ex}$ and the ICT-induced quasi-free electrons ${N}_{T}^{el}$. The decay of excitons excite the valence band electrons in graphene via IET with a transfer time of *τ*_IET_ (Fig. 4f). On the other hand, the arrival of ICT-induced electrons transiently shifts the VBs of WSe_2_ (green curve in Fig. 4a) which therefore represents the dynamics of ${N}_{T}^{el}$ as discussed before. We assume VB1 and VB2 shift in the same way (fitting details see SI). The VB1 shifting shows a time delay of ~65 fs compared to the CB signal, evidencing the occurrence of interlayer hot electron injection after photoexcitation. The population of ${N}_{T}^{el}$ subsequently relaxes back to *K*_Gr_, refilling the excited states of graphene (Fig. 4h). From the graphene side, the photoexcited hot electrons ${N}_{Gr}^{el}$ (red curve in Fig. 4b) could either scatter to conduction bands of WSe_2_ or relax by interband decay channels in graphene. Therefore, the relaxation of ${N}_{Gr}^{el}$ could be characterized with the charge transfer time of *τ*_ICT_ and a decay time of ${\tau }_{Gr}^{el}$. The deep valence band holes ${N}_{Gr}^{h}$ (blue curve in Fig. 4b) are populated by exciton energy transfer on a time scale of *τ*_IET_, which would relax back to the Fermi level with a lifetime of ${\tau }_{Gr}^{h}$.

The complete dynamics across the interface can be described with a set of coupled rate equations based on a multi-level scheme (details see SI). By numerically solving the rate-equation model, we disentangle the dynamics of IET and ICT. Our global fit describes the data well and yields the transfer times of *τ*_IET_ = 67 ± 7 fs and *τ*_ICT_ = 118 ± 18fs. The lifetimes of electrons and IET-populated hot holes in graphene are simultaneously extracted as ${\tau }_{Gr}^{el}=84\pm 7\,{{{{{{{\rm{fs}}}}}}}}$ and ${\tau }_{Gr}^{h}=7\pm 4\,{{{{{{{\rm{fs}}}}}}}}$. Combining all our observations and analysis of the energy-momentum dynamics in WSe_2_ and graphene, we summarize the interfacial phenomena governing the non-equilibrium behavior of our heterostructure: first, the optical pump generates excitons in WSe_2_ and quasi-free carriers in graphene (Fig. 4e). Following photoexcitation, the exciton annihilation excites deep valence electrons in graphene via an IET process (Fig. 4f, g). Simultaneously, hot electrons in graphene are injected to the conduction bands of WSe_2_ via ICT which transiently shift the valence bands of WSe_2_ (Fig. 4h).

## Discussion

To elucidate the interfacial coupling mechanism at play in our experiment, in particular the observed ultrafast energy transfer rate, we perform microscopic calculations of three types of IET mechanisms: Meitner-Auger, Förster, and Dexter energy transfer. The interlayer MA process is described by the dipole-monopole energy transfer from excitons to valence band excitation, schematically shown in Fig. 4e. The photoexcited hot holes in graphene quickly relax and distribute below *E*_*F*_ near a transient chemical potential ${\mu }_{Gr}^{h*}$. This allows an MA-type transition from the deep valence band to the hot hole vacancy by absorbing the exciton energy. The microscopically calculated transfer rate is plotted as a function of **Q** in Fig. 4c with different transient chemical potentials for the hole distributions ${\mu }_{Gr}^{h*}$. When the hole vacancy is located around ${\mu }_{Gr}^{h*}=-0.3\,{{{{{{{\rm{eV}}}}}}}}$, the maximum transfer rate reaches *Γ*_IET_ = 2.4 meV, corresponding to a *τ*_IET_ = 270 fs transfer time. The MA-type IET process could describe the observed energy-momentum distribution of intraband transition of valence electrons in a reasonable quantitative agreement with the extracted transfer rate. As the transient chemical potential is subject to the doping level of graphene, we also calculate the MA-type IET with n-doped graphene by artificially increasing the Fermi energy. The IET-induced intraband transition in the valence bands is suppressed with decreased photo-generated hole vacancies. However, the intrinsically doped electrons above the Dirac point enable the MA-type IET in the conduction bands (details see SI).

Another IET mechanism is Förster energy transfer (Fig. 4f). The energy of the exciton excites an interband transition from valence bands to above Dirac point via the dipole-dipole coupling^55^. In contrast to the MA-type IET process, the interband excitation via Förster-type energy transfer populates the conduction bands of graphene above the Fermi level, independent of the photon-induced hot carriers distribution. The coupling strength is explicitly evaluated (for derivation, see SI) and determined by the momentum **Q** and interlayer distance *d*. The strong exciton oscillator strength and intrinsic in-plane exciton dipole moment in many 2D materials favor the Förster-type IET^56^. However, the calculated transfer rate is only 0.08 meV (a transfer time of ~8.1 ps), even assuming a tightly stacked heterostructure with interlayer distance of *d* = 0nm (Fig. 4d). Our calculations reveal that the IET process preferably excites an intraband rather than an interband transition. The experimentally observed energy-momentum distribution of excited-state hot holes supports this conclusion. To further distinguish the MA- and Förster-type IET, we calculate the transfer rates of these two mechanisms as a function of layer distance, and identify the distinct layer distance dependence (details see SI). In addition, we also performed calculations of Dexter-type IET (Fig. 4g), in which scenario the electron and hole components of excitons in WSe_2_ scatter to the graphene layer simultaneously. However, due to the small wavefunction overlap and the finite momentum distance between ${{{{{{{{\rm{K}}}}}}}}}_{{{{{{{{{\rm{WSe}}}}}}}}}_{{{{{{{{\rm{2}}}}}}}}}}$ and K_Gr_, we found a very weak Dexter-type interlayer coupling strength, more than three orders of magnitude smaller compared to the other two mechanisms (see SI). Compared with Förster- and Dexter-type IET, the calculated transfer time of MA-type energy coupling is the closest to our experimental results. We can thus identify the MA-type conversion of excitons in WSe_2_ to intraband excitations in graphene as the dominant IET mechanism.

In this work, we provide a detailed microscopic picture of interfacial charge and energy transfer processes in photoexcited ML-WSe_2_/graphene heterostructures. Optical excitation of electrons in graphene leads to interlayer charge transfer of quasi-free electrons from the graphene layer to the K and Q valleys of the semiconductor’s conduction bands on a time scale of ~50 fs. In contrast, excitons in WSe_2_ decay through an interfacial Meitner-Auger energy transfer process with a time constant of ~70 fs. This previously unidentified process is governed by interlayer dipole-monopole interactions leading to annihilation of an exciton in WSe_2_ and non-vertical intraband excitations in graphene. The momentum of the electron-hole pair in graphene originates from the finite center of mass momentum of the hot excitons in WSe_2_. The interfacial Meitner-Auger mechanism is found to dominate the energy transfer process over established mechanisms like Förster- and Dexter-type transfer. This mechanism results in transient hole distributions as low as 2 eV below the Dirac points. These observations enrich the physical toolbox for designing van der Waals heterostructures and might be utilized in hot-carrier photovoltaic device concepts to harness the ultrafast and efficient carrier transfer processes at interfaces^57^.

## Methods

### Time- and angle-resolved photoemission spectroscopy

We used a 500 kHz tabletop femtosecond optical parametric chirped pulse amplification (OPCPA) laser system operating at a center wavelength of 800 nm and delivering average power up to 15 W. The high harmonic generation is produced in a vacuum chamber by tight focusing (10 μm) the second harmonic (400 nm) of the OPCPA fundamental on a thin and dense argon gas jet. We select the photons ~21.7 eV (110 meV FWHM bandwidth) as the probe arm for trARPES experiment^27^. Concerning the pump arm, we used two different beams for this study. One pump beam is directly obtained from the OPCPA (800 nm, FWHM = 35 fs) and another one is the residual power of the compressed fiber amplifier (1030 nm, FWHM = 200 fs). The pump and probe beams are coupled into an ultra-high-vacuum (UHV) chamber and spatially overlapped at the sample position which is controlled by a six-axis manipulator (Carving, SPECS GmbH). The main UHV chamber is equipped with a unique combination of a hemispherical electron energy analyzer (PHOIBOS150, SPECS GmbH) and time-of-flight (ToF) momentum microscope (METIS1000, SPECS GmbH)^28^. On the one hand, the hemispherical analyzer, which can work in a multi-electrons per laser shot regime, provides high statistic energy/momentum cuts along a given momentum direction, as shown in Fig. 3. On the other hand, the momentum microscope allows for efficient, parallel, momentum-resolved detection of the full photoemission horizon from the surface as shown in Fig. 1b and Fig. 2a–e. All the experiments are performed at room temperature.

### ML-WSe_2_/ML-graphene vdW heterostructure fabrication

Monolayer graphene on SiC (Si-terminated surface) was grown using the well-established recipe of sublimation growth at elevated temperatures in an argon atmosphere^4^. Note that, on SiC^58^, the graphene monolayer resides on top of a $(6\sqrt{3}\times 6\sqrt{3})$R30^∘^ reconstructed carbon buffer layer that is covalently bound to the SiC substrate. WSe_2_ films were grown on the thus prepared MLG/SiC substrates via hybrid-pulsed-laser deposition (hPLD) in ultra-high vacuum^35^. Pure tungsten (99.99%) was ablated using a pulsed KrF excimer laser (248 nm) with a repetition rate of 10 Hz, while pure selenium (99.999%) was evaporated from a Knudsen cell at a flux rate of around 1.5 Å/s as monitored by a quartz crystal microbalance. The deposition was carried out at 450 °C for 6 h, followed by two-step annealing at 640 °C and 400 °C for 1 h each.

### DFT band structure calculations

We performed density functional theory (DFT) calculation of suspended ML WSe_2_ and graphene with the projector augmented wave code GPAW^59^ using GLLB-SC xc-functional, separately. The GLLB-SC is an orbital-dependent exact exchange-based functional including the spin-orbital coupling^60^. The relaxed lattice constant of WSe_2_ is *a* = 3.25 *Å*. We sample the Brillouin Zone with a (15 × 15 × 1) k-point mesh, and set the cutoff energy for the plane-wave expansion at 600 eV. The bandgap is adjusted to fit our data. The calculated band structures of both materials are superimposed on each other and shown in Fig. 2a.

### Supplementary information

Supplementary Information

Peer Review File

## Data Availability

The trARPES data generated in this study have been deposited in the Zenodo database under accession code 10.5281/zenodo.8210835.

## Code availability

The source code for the trARPES data analysis is available on GitHub (https://github.com/mpes-kit). The data processing details are well described in ref. ^61^.
