# Supplementary material for: Observation of ultrafast interfacial Meitner-Auger energy transfer in a Van der Waals heterostructure
Source: Nat Commun. 2023 Aug 19;14:5057. doi: 10.1038/s41467-023-40815-8 (PMC10439896; doi:10.1038/s41467-023-40815-8)
Supplement: Supplementary file 1 — Supplementary Information [file 41467_2023_40815_MOESM1_ESM.pdf]

Supplementary Information for: Observation of ultrafast interfacial Meitner-Auger energy transfer in a van der Waals heterostructure

Shuo Dong[1,2*], Samuel Beaulieu[1,3], Malte Selig[4], Philipp Rosenzweig[5], Dominik Christiansen[4], Tommaso Pincelli[1], Maciej Dendzik[1,6], Jonas D. Ziegler[7,8], Julian Maklar[1], R. Patrick Xian[1,9], Alexander Neef[1], Avaise Mohammed[5], Armin Schulz[5], Mona Stadler[10], Michael Jetter[10], Peter Michler[10], Takashi Taniguchi[11], Kenji Watanabe[12], Hidenori Takagi[5,13,14], Ulrich Starke[5], Alexey Chernikov[7], Martin Wolf[1], Hiro Nakamura[5,15], Andreas Knorr[4], Laurenz Rettig[1*] & Ralph Ernstorfer[1,16*]

[1]*Fritz-Haber-Institut der Max-Planck-Gesellschaft, Faradayweg 4-6, 14195 Berlin, Germany*

[2]*Beijing National Laboratory for Condensed Matter Physics, Institute of Physics, Chinese Academy of Sciences, Beijing 100190, China*

[3]*Université de Bordeaux - CNRS - CEA, CELIA, UMR5107, F33405, Talence, France*

[4]*Nichtlineare Optik und Quantenelektronik, Institut für Theoretische Physik, Technische Universität Berlin, 10623 Berlin, Germany*

[5]*Max Planck Institute for Solid State Research, 70569 Stuttgart, Germany*

[6]*Department of Applied Physics, KTH Royal Institute of Technology, Hannes Alfvéns väg 12, 114 19 Stockholm, Sweden*

[7]*Institute of Applied Physics and Würzburg-Dresden Cluster of Excellence ct.qmat, Technische Universität Dresden, 01062 Dresden, Germany*

[8]*Photonics Laboratory, ETH Zürich, 8093 Zürich, Switzerland*

[9]*Department of Statistical Sciences, University of Toronto, 700 University Avenue, Toronto, ON*

M5G 1Z5, Canada

[10]*Institute of Semiconductor Optics and Functional Interfaces, Research Center SCoPE and IQST, University of Stuttgart, 70569 Stuttgart, Germany*

[11]*International Center for Materials Nanoarchitectonics, National Institute for Materials Science, 1-1 Namiki, Tsukuba 305-0044, Japan*

[12]*Research Center for Electronic and Optical Materials, National Institute for Materials Science, 1-1 Namiki, Tsukuba 305-0044, Japan*

[13]*Department of Physics, University of Tokyo, 113-0033 Tokyo, Japan*

[14]*Institute for Functional Matter and Quantum Technologies, University of Stuttgart, 70569 Stuttgart, Germany*

[15]*Department of Physics, University of Arkansas, Fayetteville, Arkansas 72701, USA*

[16]*Institut für Optik und Atomare Physik, Technische Universität Berlin, 10623 Berlin, Germany*

This file includes:

Supplementary Notes

Supplementary Methods

Supplementary Equation (1) to (88)

Supplementary Fig.1 to Supplementary Fig.14

Supplementary Table 1

References

Supplementary Notes

Characterization of the ML-WSe_2/graphene heterostructure

Raman measurements were carried out at room temperature using a 532 nm laser with a power of 1 mW and a spot size of 5 to 10 μm. As shown in Supplementary Fig. 1**a**, ML-WSe_2 is confirmed by an intense peak at 250 cm^{-1} which comes from essentially degenerate A_{1g} and E_{2g} lattice vibration modes[1,2]. Photoluminescence (PL) measurements are performed using another system with a 532 nm excitation laser, power of 1 mW and spot size of 1 μm at room temperature. In Supplementary Fig. 1**b**, ML-WSe_2 on graphene presents two weak PL peaks (778 nm and 914 nm) only slightly above the background. The peak at 778 nm is close in energy to the A-exciton transition energy[3,4]. The origin of the peak at higher wavelength is unknown, and may come from the existence of in-gap defect states. The weakness of the PL signals is consistent with the quenching of PL known to occur for ML-TMDCs adjacent to graphene[5].

The sample is protected by the Se capping layer before sending to our lab. After introducing the sample into our ultrahigh vacuum (UHV) photoemission end-station, we have annealed the sample for 15 minutes at 400°C through direct current heating to remove the Se capping. After annealing, we recorded a low energy electron diffraction (LEED) pattern with the incident beam energy of 95 eV, to verify the surface cleanliness and ordering (Supplementary Fig. 1**c**). The six outer sharp LEED spots come from the bottom ML graphene layer (yellow box) and the inner six arc-shaped diffraction spots originate from the top ML-WSe_2 layer (red box). The occurrence of a well-oriented hexagonal pattern of WSe_2 spots aligned to the graphene pattern attests the epitaxial

nature of our heterostructure and single-domain lattice orientation. A certain level of strain-induced misalignment between nanoislands of WSe_2 with respect to the graphene layer is evident from the azimuthal widths of the diffraction spots.

Our sample exhibits areas without WSe_2, since the top WSe_2 layer consists of spatially uniform small islands with some distance between the islands. However, the relevant processes discussed in our manuscript, *i.e.*, both interlayer hot electron injection with 1.2 eV pump and Meitner-Auger type interlayer energy transfer with 1.55 eV pump, are based on the photoemission signals from the heterostructure, but will not occur at an isolated graphene or WSe_2 layer. For example, the observation of the excited state population at WSe_2 with below-bandgap excitation is not possible for an isolated monolayer WSe_2. With near-resonant pump, the deep-lying holes would not be excited in a pure graphene sample. Therefore, the signal discussed in our work is based on the heterostructure area.

Additionally, the doping level of epitaxial graphene is different from the pristine graphene without WSe_2. We discuss possible reasons for this observation: static charge (electron) transfer from graphene to WSe_2, or the decrease of electrons at the graphene/SiC interface with W or Se intercalation as the referee suggested. In the latter case that a substantial part of the Se or W elements is intercalated below the graphene layer, the intensity of the diffraction pattern of SiC, *i.e.*, 6×6 spots around the graphene (10) and diamond-shape distributed spots around $\sqrt{3}\times\sqrt{3}$ SiC, would be dramatically reduced. As we observed a clear SiC pattern in our LEED image (Supplementary Fig.1**c**), this scenario seems unlikely. Second, we would expect graphene bilayer

growth with Se or W intercalation, which is also not observed. Finally, there is no core level energy shift of SiC peaks after the growth of WSe_2[6], which demonstrates the band alignment at the graphene/SiC interface (band bending of p-n junction) is not influenced by the top layer. These observations rule out a significant contribution of W or Se intercalation to the graphene's doping level. Therefore, we conclude on a static charge transfer from graphene to WSe_2 is the major contribution to the modification of the doping of graphene.

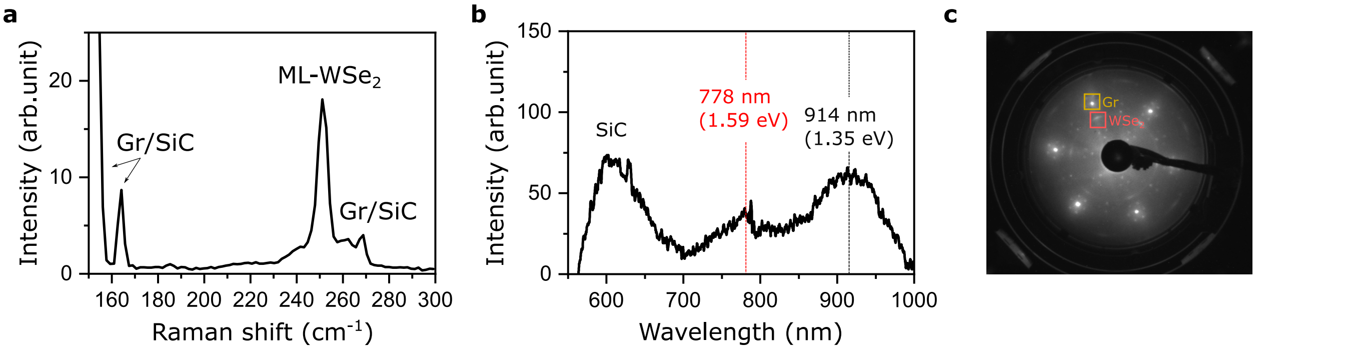

Supplementary Fig. 1: Optical characteristics and surface analysis of the heterostructure sample. **a**, Raman measurement of ML-WSe_2/graphene (Gr) at room temperature. An intense peak at 250 cm^{-1} belongs to ML-WSe_2, whereas other peaks belong to the Gr/SiC substrate. **b**, Photoluminescence measurement of the ML-WSe_2/Gr heterostructure. The peak originating from the A-exciton of ML-WSe_2 is marked with the dashed red line. **c**, LEED pattern of the ML-WSe_2/Gr heterostructure at 95 eV after annealing.

Delayed population rises at K_{WSe_2} and Q_{WSe_2} with the below-bandgap excitation

Upon 1.2 eV excitation, the time trace at the K_{Gr} points shown in Fig.2**f** is by fitting with a

single exponential decay function convolved with the instrument response function (IRF), $I(t) = H(t-t_0) \times (A \cdot \exp(-(t-t_0)/\tau) + C) \otimes \mathrm{IRF}$. Here, $H(t)$ is the Heaviside step function, IRF is Gaussian envelope function, A is the amplitude and C is the offset. In contrast, as the $\mathrm{K_{WSe_2}}$ and $\mathrm{Q_{WSe_2}}$ valleys are populated by interlayer charge transfer (ICT) processes, the corresponding time traces are fitted with an exponential growth function to describe the ICT process adding with a single exponential decay for relaxation. The delayed population rise between graphene and WSe_2, $\Delta t = 51 \pm 9$ fs, is obtained by taking the time difference between the peak at $\mathrm{K_{Gr}}$ and the time delay when the population at the $\mathrm{K_{WSe_2}}$ valley reaches its maximum.

Exclusion of two/multiple-photon absorption in WSe_2

Upon 1.2 eV excitation, we could rule out the two/multiple-photon absorption of WSe_2 based on three experimental observations. First, we find no excited state population of WSe_2 in the energy range higher than the conduction band minimum from 1.0 to 2.5 eV as shown in Supplementary Fig.2**a**. The valence band maximum of WSe_2 is identified at $E - E_F = -1.1$ eV by the EDC analysis in Fig.**3d** (main text). In the scenario of two-photon absorption, the photo-induced carriers would be directly populated at $E - E_F = 1.3$ eV. This direct optical excitation is not observed in our measurement. The three-photon absorption, corresponding to the excitation at $E - E_F = 2.5$ eV, is more difficult to happen and is neither observed. Second, the delayed population rise in $\mathrm{K_{WSe_2}}$ and $\mathrm{Q_{WSe_2}}$ valleys compared to the rise of hot carriers in graphene, as shown in Fig.2**f** (main text), is strong evidence of the absence of the direct optical excitation in WSe_2. The hot carrier rise in graphene demonstrates the arrival of the pump pulse. However, the excited-state population of the

WSe_2 layer appears ∼50 fs after the pump excitation. Finally, by integrating the photoemission intensity, we present the population dynamics of electron (ROI_1 in Supplementary Fig.2**a**) and hole components (ROI_2 in Supplementary Fig.2**a**) in WSe_2 upon 1.2 eV excitation. Different from the hot electron dynamics (blue curve in Supplementary Fig.2**b**), the intensity of holes (red curve in Supplementary Fig.2**b**) stays constant without any signature of optical excitation. The absence of hole dynamics could also be explained by an ultrafast interlayer hole transfer on a time scale much shorter than our temporal resolution. However, combined with the first two observations, we exclude the occurrence of two/multiple photon absorption. With near-resonant excitation, the two-photon absorption is also excluded since no excited-state population is found at $E - E_F = 2.0$ eV, which corresponds to the energy of excited-state electrons by absorbing two pump photons.

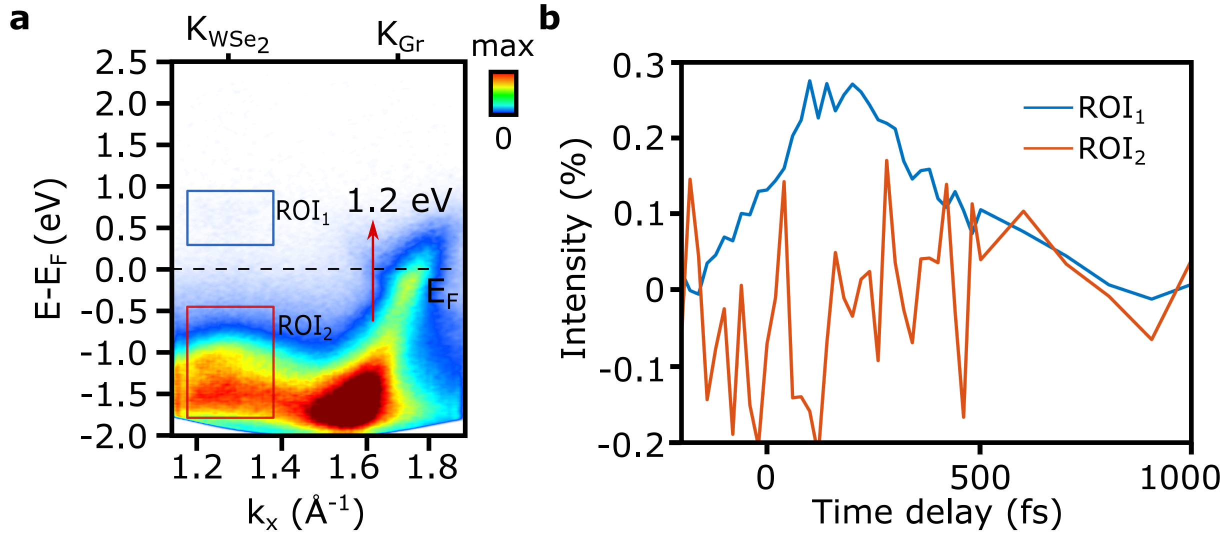

Supplementary Fig. 2: Carrier dynamics upon the below-bandgap excitation a, Two dimensional energy-momentum cut with 1.2 eV pump. **b**, By integrating the photoemission intensity in ROI_1 and ROI_2 in **a**, the population dynamics of electrons (blue curve) and

holes (red curve) are extracted, respectively. The conduction band of WSe_2 is filled via interlayer hot electron injection. The population of holes stay constant without the signature of optical excitation. The time traces are normalized to total photoemission intensity at negative time.

Identification of the Fermi level and distribution of excited-state carriers at $\mathrm{K}_{\mathrm{WSe}_2}$

In both experiments, the energy axis calibration has been performed using the position of the Fermi level of graphene, which is obtained from the energy distribution curve (EDC) at the Dirac point of graphene (Supplementary Fig. 3**a** and **d**). Before optical excitation, the EDCs at the K_{Gr} point (Supplementary Fig. 3**b,e**) are fitted with a Fermi-Dirac distribution function at 300 K convolved with the IRF ($\sim$150 meV FWHM) determined by the energy resolution of the spectrometer and the bandwidth of the probe pulses[7]. The chemical potentials are set to be zero for both experimental conditions to remove the XUV-probe-induced space charging effect in each measurement. The EDCs in Supplementary Fig. 3**b,e** are integrated over a momentum window, $\Delta k = 0.1\ \text{Å}^{-1}$ and selected at negative time delay. Based on the energy reference obtained from the Fermi level fits, the energy positions of the conduction band minima at the $\mathrm{K}_{\mathrm{WSe}_2}$ point can be obtained from EDCs showing the excited state carrier distributions of WSe_2 upon resonant and off-resonant excitation as displayed in Supplementary Fig. 3**c** and **f**, respectively. The photoemission intensity has been normalized by the total electron count of the spectrum. The energy difference of the carrier distribution, more specifically, the smaller kinetic energy of excited carriers with 1.55 eV pump, arises from the exciton formation upon the resonant excitation. By fitting with a single Gaussian

lineshape on top of a empirical second-order polynomial background, the energies of excited-state carriers are extracted as 0.63 eV upon the resonant A-exciton excitation (Supplementary Fig. 3**c**) and 0.73 eV upon the below-bandgap excitation (Supplementary Fig. 3**f**). The background has been removed in the main text (side figures in Fig.3**a-b**).

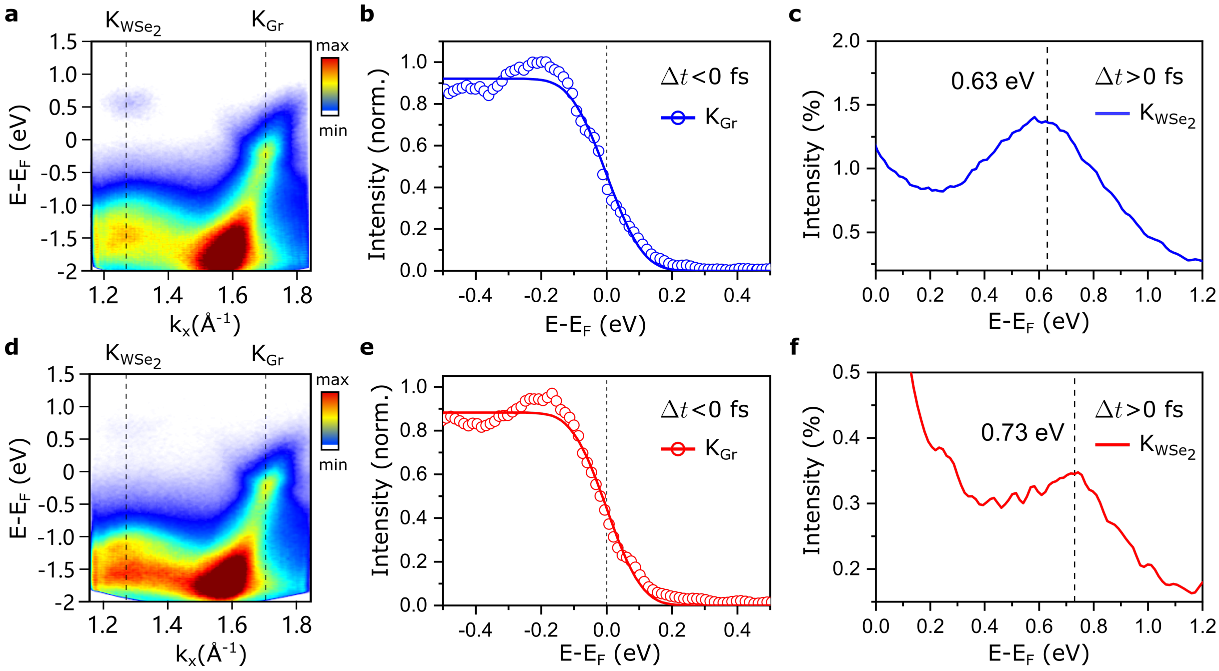

Supplementary Fig. 3: Fermi level calibration. a,d, The 2D photoemission intensity spectra as a function of energy and momentum at time zero with 1.55 eV and 1.2 eV pump, respectively. **b,e**, EDCs of K_{Gr} at negative time delay fitted with a Fermi-Dirac distribution convolved with the energy IRF for 1.55 eV and 1.2 eV pump, respectively. The chemical potentials are aligned to zero by rigidly shifting the energy axis in both cases. **c, f** EDCs at K_{WSe_2} integrated within the first 100 fs obtained with 1.55 eV and 1.2 eV pump, respectively. The dashed lines represent the center of the excited-state carrier distributions extracted by a fitting procedure (see text).

Identification of the Dirac point energy

To identify the energy position of the Dirac point, we selected an energy-k_y cut ($\Delta t < 0$ fs) at Dirac point and along the green dashed line in Supplementary Fig. 4**a**. The small titling angle allows us to see both valence bands clearly in Supplementary Fig. 4**b**. We track the graphene valence band dispersion by fitting the momentum distribution curves of occupied bands with two Voigt lineshape functions. Then, each graphene valence band is fitted to a linear dispersion and the Dirac point is estimated at the intersection of two lines (red and black), $E - E_F = -0.10 \pm 0.05\,\text{eV}$, in a reasonable agreement with the previous characterization of similar heterostructures[6]. This energy/momentum cut is different than the one presented in the main text (Fig.3) along the Γ-K direction, which is featured by the suppression of one side of the cone due to photoemission matrix element effects (sublattice interference)[8]. In the main text, we choose this cut direction because it allows us to clearly resolve the excited state dynamics from both layers.

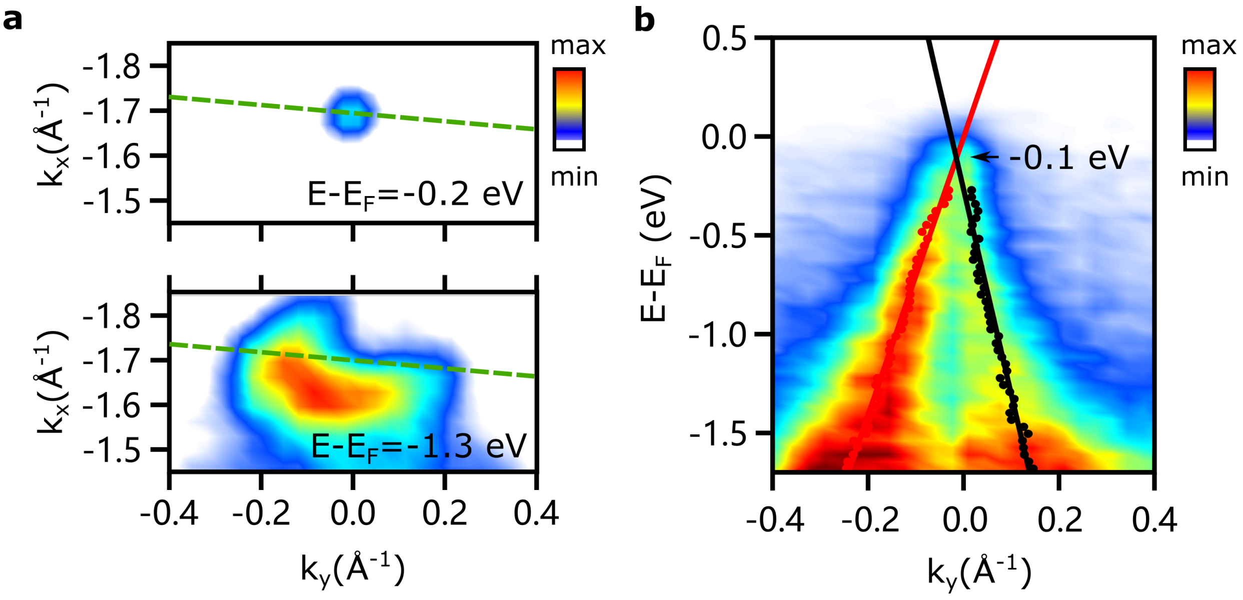

Supplementary Fig. 4: Experimental determination of the Dirac energy. **a**, 2D momentum distribution map $I(k_x, k_y)$ at $E - E_F = -0.2$ eV and $E - E_F = -1.3$ eV. At the boundary of the Brillouin zone, it shows the quasi-triangular-shaped π band of graphene. **b**, Energy/momentum cut $I(E, k)$ along the green dash line in **a** showing the conical band dispersion of graphene. Red and black markers indicate the band positions extracted from momentum distribution curve fits. Lines are linear fits of the band positions, yielding the energy position of the Dirac point of $E_D = -0.1 \pm 0.05$ eV.

Identification of the Fermi velocity

The Fermi velocity along Γ-K direction is extracted as $v_F = (1.8 \pm 0.1) \cdot 10^6\ m/s$ as shown in Supplementary Fig. 5 using the same band dispersion tracking method in the above paragraph. The energy/momentum spectrum is selected at negative time delay ($\Delta t < 0$ fs). The Fermi velocities in the direction perpendicular to Γ-K with a slightly tilting angle (Supplementary Fig. 4**b**) are $v_F = (1.1 \pm 0.1) \cdot 10^6\ m/s$ (red) and $v_F = (1.5 \pm 0.1) \cdot 10^6\ m/s$ (black), respectively. The Fermi velocity of graphene has been found to to be in the range of $1 \cdot 10^6$ to $3 \cdot 10^6$ m/s, depending on the dielectric constant of the environment[9]. For epitaxially grown heterostructures, the dielectric constant of the embedding graphene layer between the bottom substrate and top TMDC layer could be modified by the coverage sizes of TMDC layer and substrate material, as the dielectric constant is determined by $\epsilon = (\epsilon_{top} + \epsilon_{substrate})/2$. At the same time, the Fermi velocity is also sensitive to the graphene's doping level[10]. We would like to note that because of the steep band dispersion of the graphene band, the momentum of transiently excited intraband electron-hole pairs

is small. Therefore, it requires a relative small excitonic COM momentum $\mathbf{Q}$ to fulfill energy and momentum conservation, which favors the IET process.

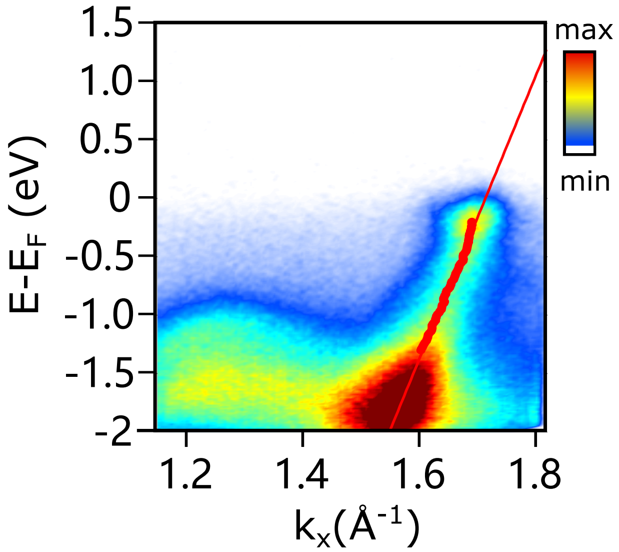

Supplementary Fig. 5: Experimental determination of the Fermi velocity. The energy/momentum cut $I(E,k)$ along Γ-K direction with band positions (red markers) and the linear fit of the band dispersion (red line).

The near-unity efficiency of IET

To estimate the efficiency of energy transfer, we performed the same measurement (1.55 eV excitation) on bare ML-WSe_2, which is prepared by scotch-tape exfoliation and transferred on top of thin hexagonal boron nitrid (hBN) with conductive TiO_2 substrate (Supplementary Fig. 6**a**). The efficiency of the energy transfer process is commonly defined by the lifetime of the 'donor' material (here WSe_2) with and without the 'acceptor' material (here graphene) as: $\eta_{ET} = (\tau_{ML} - \tau_{hetero})/\tau_{ML}$, where τ_{ML} represents the exciton lifetime of the bare ML-WSe_2 and τ_{hetero} is the

exciton lifetime in the WSe_2/graphene heterostructure. The excited-state population dynamics at the $\mathrm{K}_{\mathrm{WSe}_2}$ valley within each system are presented in Supplementary Fig. 6**b**. The lifetimes of the ML sample $\tau_{ML} = 1616 \pm 345$ fs is extracted by fitting with an exponential decay function convolved with the IRF. The exciton lifetime of the heterostructure $\tau_{hetero} = 67 \pm 7$ fs is obtained by solving the system of rate equations as described in the main text. Thus, we obtain for the interlayer energy transfer efficiency, $\eta_{ET} = 96 \pm 1\%$. This near-unity transfer efficiency is supported by the underlying conservation of energy and momentum. We note that the different sample fabrication methods of the bare ML and heterostructure may have influence on the exciton lifetime. However, picosecond to sub-nanosecond exciton lifetimes in ML samples, consistent with our observations, have been reported for samples fabricated with various methods[11,12]. Therefore, we believe that the comparison with the exciton lifetimes in the bare ML WSe_2 sample provides a reasonable estimate of the transfer efficiency.

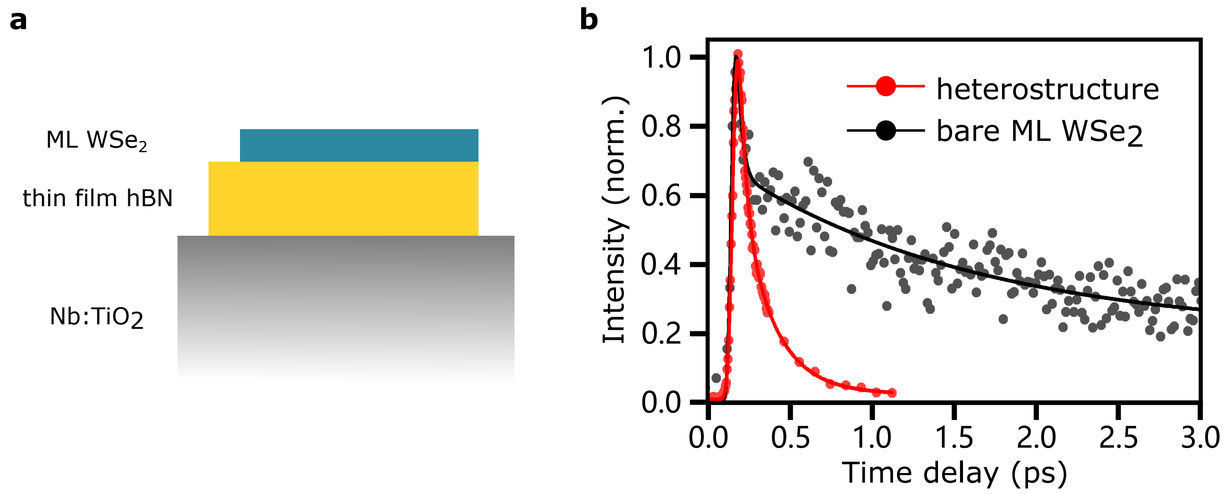

Supplementary Fig. 6: Estimation of the IET efficiency. **a**, Schematic of the bare ML-WSe_2 sample (blue slab) with the bottom hBN layer (yellow) mounted on a Nb:TiO_2

substrate (grey). **b**, Time traces of the excited-state carriers at the $\mathrm{K}_{\mathrm{WSe}_2}$ valleys of the bare ML sample (black) and heterostructure (red) sample, respectively.

Valence bands shifting and broadening effects

After photoexcitation, we observe shifting and broadening effects of the WSe_2 valence band as shown in the 2D difference spectrum (Fig. 3**c**) and EDCs at $\mathrm{K}_{\mathrm{WSe}_2}$ (Fig. 3**d**). To extract the transient lineshape, we fit the EDC of the top two VBs (VB1 and VB2) with two Gaussian functions on top of an empirical second-order polynomial background (BG), $I(E) = A_1 \cdot \exp(-\frac{(E-E_1)^2}{2\omega_1^2}) + A_2 \cdot \exp(-\frac{(E-E_2)^2}{2\omega_2^2}) + \mathrm{BG}$, where E_1, E_2 are peak positions and ω_1, ω_2 are the peak width. Supplementary Fig. 7**a-d** present representative fitting results at four time delays, $\Delta t < 0$ fs, $\Delta t = 0$ fs, $\Delta t = 200$ fs and $\Delta t = 1000$ fs. Because of the large spectral overlap between VB1 and VB2, the fitting is performed with the same shifting, $\Delta E_1(t) = \Delta E_2(t)$, and broadening parameter , $\Delta\omega_1(t) = \Delta\omega_2(t)$, for the two peak functions, assuming the VBs respond to the interfacial coupling and the excitation-induced modification in the same way. The extracted time dependent peak shift and linewidth parameter are shown in Supplementary Fig. 7**e** and **f**, respectively. The error bars are confidence interval of the band fitting process. The band shifting reflects the electronic band gap renormalization due to the ICT-induced hot electrons[13,14]. Thus, a relative time delay can be observed compared with the excited-state population dynamics in the conduction bands. In contrast, the linewidth is a measure of the photohole self-energy, which depends on the many-body interactions with photoexcited carriers and phonons[15]. It follows more closely the transient of overall excited carriers in the system (grey dashed curve in Supplementary Fig. 7**f**).

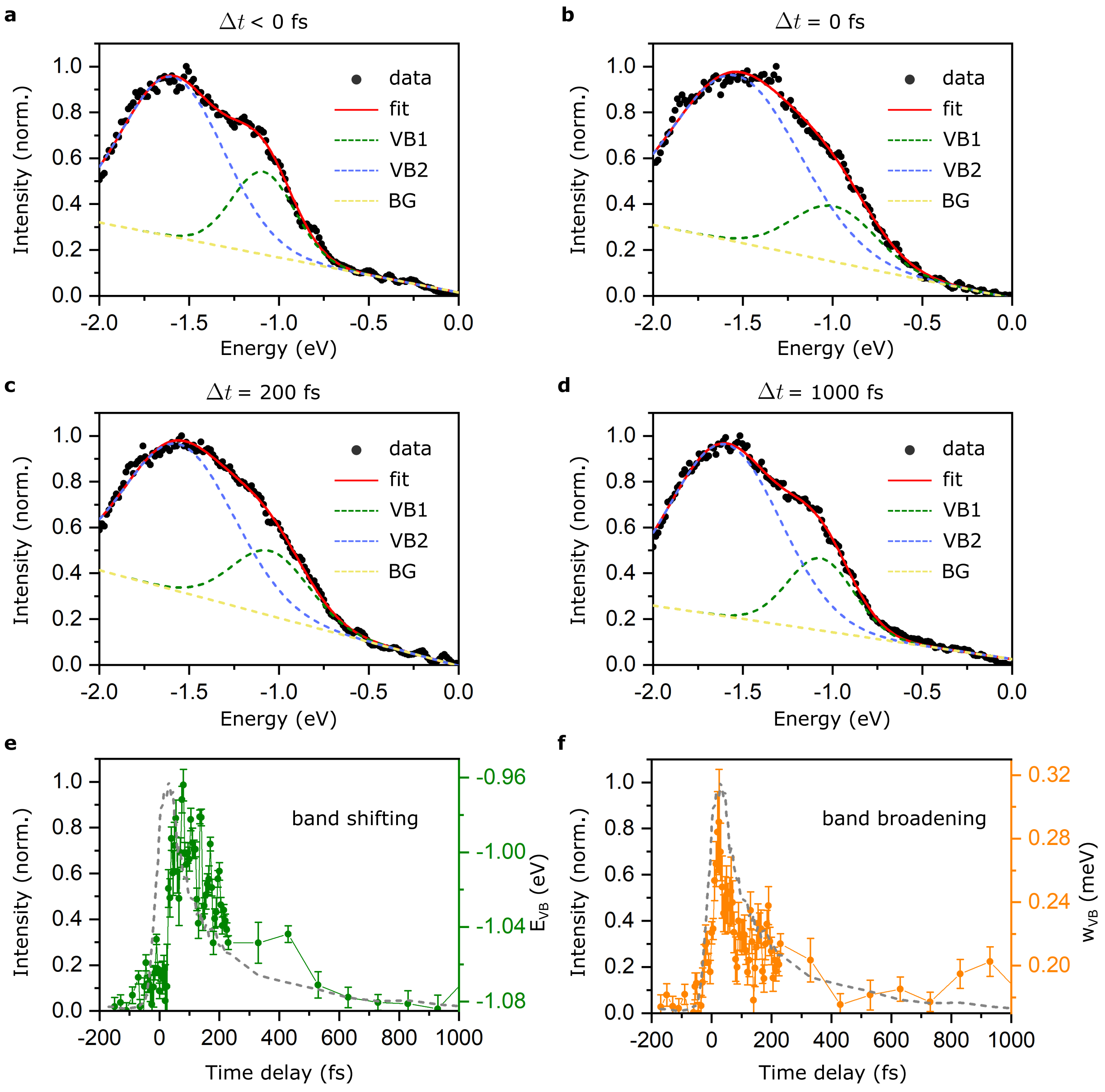

Supplementary Fig. 7: Transient lineshape of the valence bands of WSe_2. a-d, The EDCs at the $\mathrm{K}_{\mathrm{WSe}_2}$ valley present the spectral features of the first two valence bands at selected time delays, $\Delta t < 0$ fs, $\Delta t = 0$ fs, $\Delta t = 200$ fs and $\Delta t = 1000$ fs. The EDCs are fitted with two Gaussian functions describing VB1 (green dashed curve) VB2 (blue dashed curve). The second-order polynomial background is shown as yellow dashed

lines. **e**, Transient peak position of VB1 (green). **f** Peak linewidth both of VBs as function of time (yellow). The population dynamic of excited-states in the WSe_2 conduction band (grey) is shown in **e-f** as a reference. The error bars are confidence interval of band fitting.

Meitner-Auger type IET-induced hot electrons near the Fermi level

The Meitner-Auger-like interlayer energy transfer involves an intraband excitation of the deep-lying valence electrons to the photo-generated hot holes below the Fermi level. We observe the deep-lying hot holes, as shown in Fig.**3e**(main text). However, to separate the contribution of Meitner-Auger IET induced electrons from the photo-generated hot holes of graphene near the Fermi level requires an accurate description of the intrinsic energy-momentum dynamics in the graphene layer. The carrier dynamics in graphene after photoexcitation include multiple contributions: intraband carrier-carrier scattering, interband Auger heating, phonon-mediated cooling, and Auger recombination. These components contribute to the energy-dependent dynamics along the graphene bands[16,17]. This is particularly important for states close to the Fermi level, which are subject to hot carrier accumulation from the highly excited states, carrier redistribution due to the thermalization, and carrier annihilation via Auger recombination. Together with the complication of interlayer charge and energy transfer, it is very challenging to quantitatively disentangle these dynamics with our current energy and time resolution.

Although a quantitative separation of the IET-induced electrons from the photo-generated hot holes remains difficult, some information can be gained from the energy distribution of hot

carriers below and above the Fermi level in Fig.**3e**. In particular, comparing the near-resonant and below-bandgap excitation conditions proves insightful. The ratio of the relative intensity increase above the Fermi level ($E - E_F = 0 - 1$ eV, red area) to the signal depletion below E_F ($E - E_F = -1 - 0$ eV, blue area), A_{el}/A_h can be regarded as a measure of the electron-hole imbalance in the system, and we find a significantly stronger imbalance $A_{el}/A_h = 1.6$ for near-resonate excitation, compared to $A_{el}/A_h = 1.1$ for below-bandgap excitation. This supports the occurrence of MA-type IET process. Compared with the almost identical spectral weights of electrons and holes using below-bandgap excitation, the unequal spectral areas with the 1.55 eV pump demonstrate the carrier redistribution due to the strong interlayer interactions. While we would like to note that the spectral intensities are subject to photoemission matrix elements, therefore cannot be directly interpreted as electron and hole occupations. These probe-related effects can be reasonably assumed to be identical for the two excitation conditions, thus not affecting our conclusions.

Pump fluence dependent Meitner-Auger interlayer energy transfer

The pump fluence plays an important role on the MA-type IET process, because of the deep-lying valence electrons are excited to the photo-generated hot holes. The high pump fluence generates large density of hot holes, *i.e.*, more vacancies for IET-induced intraband transition, which benefits the MA-type interlayer coupling. On the other hand, the weak pump fluence would suppress the MA-type IET process with the reduced photo-generated hole density. To check this effect, we perform the same measurement with the near-resonant excitation (1.55 eV) but weak

pump fluence, $F = 0.9\ \mathrm{mJ/cm^2}$, around half of the pump fluence applied in the manuscript, $F = 1.7\ \mathrm{mJ/cm^2}$. We find the decreased intensity of the deep-lying hole population, as shown in the Supplementary Fig.8. From the energy distribution curve (EDC) of graphene band with the weak pump fluence (blue curve), a depletion signal around $E - E_F = -1.8$ eV could be observed but weaker than that with the high pump fluence.

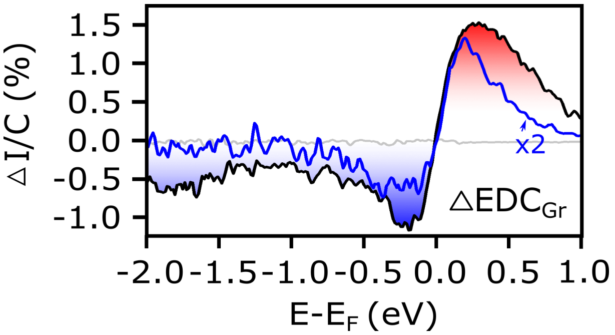

Supplementary Fig. 8: Pump fluence dependent Meitner-Auger interlayer energy transfer. The momentum-integrated spectrum of the graphene band when pumping with a weak pump fluence (blue). The EDC of graphene with the high pump fluence (black; applied in the main text) is also shown as a comparison.

Doping level dependence of interlayer energy transfer process

As we discussed in the main text, the optical excitation prepares the hot holes near the Fermi level, which enables the intraband transition under the mechanism of Meitner-Auger-type IET. The doping level of graphene plays an essential role in the energy position of the photo-generated hot holes, therefore, influencing the MA-type IET. To clarify the influence of the doping level, we calculate the Meitner-Auger type transfer rate by increasing the Fermi energy to $E_F = 0.2\,\mathrm{eV}$. As

shown in Supplementary Fig.9**a**, a dramatically suppression of the Meitner-Auger IET is observed with the transfer rate on the order of $0.06 \cdot 10^{-3}\,\mathrm{meV}$, much smaller than that of $3\,\mathrm{meV}$ based on our real sample system. Compared with the Förster-type coupling (Supplementary Fig.9**c**), the maximum of the transfer rate is even larger than that of the MA-type coupling.

However, we would like to note that MA-type IET could also happen in the conduction bands with the n-doped graphene composition. The natural doping in the graphene layer provides electrons in the conduction band, below the Fermi level and above the Dirac point. After compensating with the photo-generated hot holes near the Fermi level, the net electron population could be excited to highly excited states via the MA-type IET process. By adding the equation of motion for the conduction band electron occupation, we find a rate of the MA transfer in the conduction band as follows.

$$\Gamma_{\mathbf{Q}} = |W_{\mathbf{Q}}|^2 \frac{4}{\hbar v_F} \left(Q - \frac{E_{\mathbf{Q}}}{\hbar v_F} \right) \left(f^c_{Q - \frac{E_{\mathbf{Q}}}{\hbar v_F}} - f^c_{\mathbf{Q}} \right) \theta \left(Q - \frac{E_{\mathbf{Q}}}{\hbar v_F} \right) \tag{1}$$

with $f^c_{\mathbf{k}} = (\exp(\hbar v_F |\mathbf{k}| - \mu)/k_B T) + 1)^{-1}$ and chemical potential μ. It is similar to the transfer rate in the valence band (details see section 'Meitner-Auger-like energy transfer'), but with the electron occupation $f^c_{\mathbf{k}}$ in the conduction band.

As shown in Supplementary Fig.9**b**. the maximum transfer rate reaches $1.6\,\mathrm{meV}$. Therefore, although the Förster-type IET is faster than the intraband transition in the valence band, the MA-type IET in the conduction band is the dominant process with highly n-doped graphene. All the

calculations are performed at room temperature.

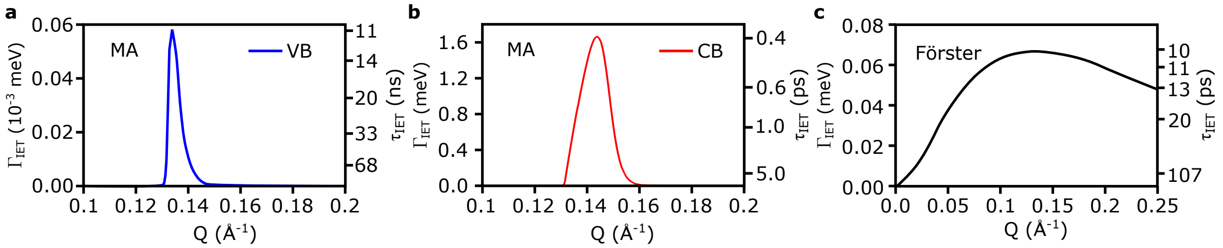

Supplementary Fig. 9: Energy transfer rate of a heretostructure with the highly n-doped graphene. a-b, The calculated Meitner-Auger transfer rate with $E_F = 0.2\,\mathrm{eV}$ in the valence band and conduction band, respectively. **c**, The calculated Förster-type transfer rate in the same condition.

<u>Band structure visualization with contrast-enhancement</u>

Fig.**1b** in the main text shows the three-dimensional data in an artistic isometric representation. The data shown in Fig.**2a-d** were treated with a contrast-enhancing algorithm to emphasize the band dispersion. The underlying dataset is the same for all these figures. In Fig.**1b**, we present the original photoemission spectrum showing the 3D band structure of the heterostructure. The linearly dispersing valence bands of graphene are emphasized by blue lines. The high symmetry points $\mathrm{K_{Gr}}$ are clearly visible in the iso-energy map shown in the top. Here, we would like to emphasize that due to the isometric representation, it might appear as though the $\Gamma_{\mathrm{WSe_2}}$ point is energetically higher than the $\mathrm{K_{WSe_2}}$ point. To illustrate this and the effect of the contrast enhancing algorithm employed for the data shown in Fig.**2**, Supplementary Fig.10 shows the energy-momentum cut of the original data at a negative time delay of $\Delta t = -400$ fs represented as

Fig.**2a** in the main text with and without the contrast enhancement. Due to matrix element effects, the photoemission intensity is inhomogeneously distributed across the Brillouin zone (Supplementary Fig.10**a**), making it difficult to visualize the band structure. Therefore, we reduced the intensity modulation in band dispersion by using a multidimensional extension of the contrast limited adaptive histogram equalization (MCLAHE) algorithm[18,19]. The MCLAHE algorithm adjusts the local image contrast, decreasing the intensity inhomogeneities without changing the band structure. The contrast-enhanced band mapping provides better visualization of the band structure, as shown in Supplementary Fig.10**b**. With the contrast-enhancement, both sides of the valence bands of graphene are visible in Fig.**2a-d**.

Meanwhile, we show energy distribution curves (EDCs) at both high symmetry points in Supplementary Fig.10**a** in the left panel, to clarify the valence band offset between the $\Gamma_{\mathrm{WSe_2}}$ and the $\mathrm{K}_{\mathrm{WSe_2}}$ point. By fitting the EDCs with Gaussian shape functions on top of an empirical second-order polynomial background, the peak position of the top valence band at $\mathrm{K}_{\mathrm{WSe_2}}$ is obtained at $E - E_F = -1.1$ eV, higher than that at $\Gamma_{\mathrm{WSe_2}}$ with the energy of $E - E_F = -1.55$ eV. The valence band maximum at the $\mathrm{K}_{\mathrm{WSe_2}}$ valley confirms the monolayer thickness of the $\mathrm{WSe_2}$ layer, as predicted by theoretical calculations[20,21] and observed in the previous ARPES measurements[22,23]. As mentioned above, the 3D band structure surface in Fig.**1b** was quarter cut open and titled forward to show the band dispersion inside the Brillouin zone in an isometric representation. This perspective drawing and the relatively intense photoemission signal at the $\Gamma_{\mathrm{WSe_2}}$ point may have caused the confusion about the energy offset between $\Gamma_{\mathrm{WSe_2}}$ and $\mathrm{K}_{\mathrm{WSe_2}}$.

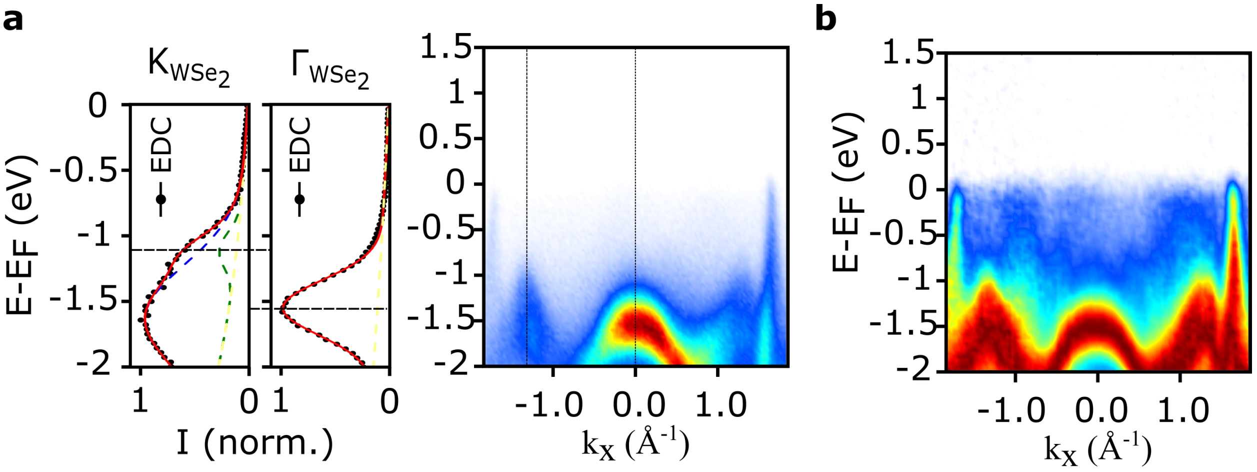

Supplementary Fig. 10: Contrast-enhanced band structure. a, The energy-momentum cut of the original photoemission signal at a negative time delay. The EDCs of the valence bands at $\Gamma_{\mathrm{WSe_2}}$ and $\mathrm{K}_{\mathrm{WSe_2}}$ are shown in the left panel figures, respectively. The energy positions of valence band maxima are emphasized with dashed black lines. **b**, The contrast-enhanced band structure from the original data.

Supplementary Methods

Characteristics of pump beams

In this work, we use two different pump beams which wavelengths are centred at 800 nm and 1030 nm, respectively (Supplementary Fig. 11). The pulse duration of 1030nm pump line is $\sim$ 200 fs FWHM, while the transform-limited pulse duration of 800 nm pump is $\sim$ 35 fs FWHM. In the measurement, the pump fluence of 800 nm is $F_{800} = 1.7\ \mathrm{mJ/cm^2}$ and that of 1030 nm is $F_{1030} = 5.3\ \mathrm{mJ/cm^2}$, with the consideration of effective pump-probe overlap profile based on the formula, $a = \frac{1}{\pi(\omega_{pump}^2+\omega_{probe}^2)}$, in the work of Harb *et al*[24]. The beam size of the pump is $\omega_{pump} = 248 \pm 20\ \mu\mathrm{m}$ and that of probe pulse is $\omega_{probe} = 80 \pm 5\ \mu\mathrm{m}$. Here, ω_{pump} and ω_{probe} are the respective beam widths of the pump and probe beams.

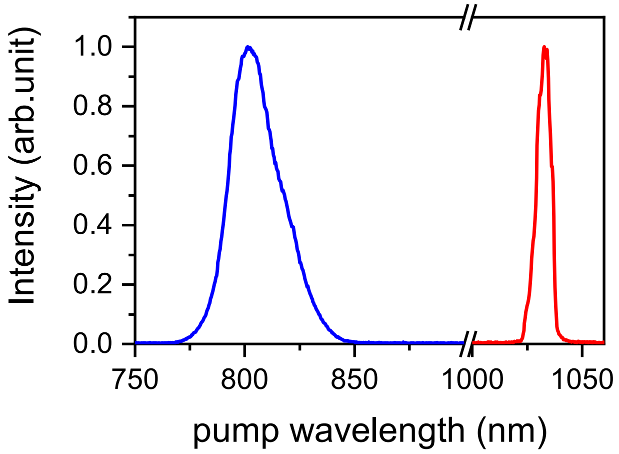

Supplementary Fig. 11: Pump spectra. Excitation spectra of the two light sources used for pumping as a function of wavelength.

Separating the interlayer charge and energy transfer by a rate equation model

As discussed in the main text, we observed the photoemission signatures of both ICT and IET upon resonant A-exciton excitation. To extract the corresponding transfer rates, Γ_{ICT} and Γ_{IET}, we develop a multi-state coupled rate equation model describing the interlayer charge and energy flow, as well as the hot carrier relaxations. In Fig.4**a**, the time trace of hot carriers in the CBM of WSe_2 (black curve) includes the dynamics of photo-generated excitons N_T^{ex} and ICT-induced quasi-free electrons N_T^{el}. The VB1 shifting (green curve in Fig.4**a**) mainly reflects the dynamic of N_T^{el}. Therefore, it provides the possibility to disentangle the dynamics of these two kinds of quasiparticles. Here, subscript *T* represents TMDC. Simultaneously, the deep valence band holes in graphene N_{Gr}^{h} are populated by the IET process and recombine with the rate Γ_h, as shown in Fig.4**b**. Finally, the dynamics of hot electrons in graphene N_{Gr}^{el} contains the ICT-induced charge flow (input and output towards WSe_2) and a decay process with the rate of Γ_{el}. With these considerations, the complete dynamics across the interface can be described with the following set of coupled rate equations:

$$\dot{N}_T^{ex} = -\Gamma_{IET} N_T^{ex} + S(t) \tag{2}$$

$$\dot{N}_T^{el} = -\Gamma_{ICT} N_T^{el} + \Gamma_{ICT} N_{Gr}^{el} \tag{3}$$

$$\dot{N}_{Gr}^{el} = +\Gamma_{ICT} N_T^{el} - \Gamma_{ICT} N_{Gr}^{el} - \Gamma_{el} N_{Gr}^{el} + S(t) \tag{4}$$

$$\dot{N}_{Gr}^{h} = -\Gamma_h N_{Gr}^{h} + \Gamma_{IET} N_T^{ex} \tag{5}$$

Here, $S(t)$ represents the optical excitation as a Gaussian-shaped pump envelope function. By numerically solving the system of coupled differential equations, and a global fit of the solution to the data, we obtain the IET transfer time $\tau_{IET} = 67 \pm 7$ fs, and the ICT transfer time $\tau_{ICT} = 118 \pm 18$ fs ($\tau = \hbar/\Gamma$). At the same time, we get the relaxation times of electrons in graphene, $\tau_{Gr}^{el} = 84 \pm 7$ fs, and that of the deep valence holes, $\tau_{Gr}^{h} = 7 \pm 4$ fs. The fitting results are shown in Fig.4**a** and **b**.

Microscopic calculation of IET mechanisms

We perform microscopic calculations of the IET process mediated by the Meitner-Auger, Förster and Dexter type mechanisms.

Meitner-Auger-like energy transfer A schematic illustration of the Meitner-Auger type (MA) interlayer transfer is depicted in the main text in Fig.4**f**. Here, an exciton in the TMDC recombines non-radiatively; its energy excites an electron deep in the valence band of graphene to states close to the Dirac point but in the valence band.

Starting point for the calculation of the MA-type interlayer coupling is the Hamiltonian

$$H_F = \sum_{\mathbf{k},\mathbf{q},\mathbf{k}',\mathbf{q}',\lambda,\lambda',\nu,\nu'} V^{\lambda\nu\nu'\lambda'}_{\mathbf{k},\mathbf{q},\mathbf{q}',\mathbf{k}'} \lambda^\dagger_{\mathbf{k}} \nu^\dagger_{\mathbf{q}} \nu'_{\mathbf{q}'} \lambda'_{\mathbf{k}'}. \tag{6}$$

As a convention, we use $\lambda^{(\prime)}$ as band indices and $\mathbf{k}^{(\prime)}$ as momenta in WSe_2 layer and $\nu^{(\prime)}$ as band indices and $\mathbf{q}^{(\prime)}$ as momenta in graphene. The appearing matrix element is formally given as

$$V^{\lambda\nu\nu'\lambda'}_{\mathbf{k},\mathbf{q},\mathbf{q}',\mathbf{k}'} = \int_{\mathbb{R}^3} d^3r \int_{\mathbb{R}^3} d^3r' \Psi^{\lambda *}_{\mathbf{k}}(\mathbf{r}) \Psi^{\nu *}_{\mathbf{q}}(\mathbf{r}') V(\mathbf{r},\mathbf{r}') \Psi^{\nu'}_{\mathbf{q}'}(\mathbf{r}') \Psi^{\lambda'}_{\mathbf{k}'}(\mathbf{r}). \tag{7}$$

The band indices in the TMD are restricted to interband transitions $\lambda \neq \lambda'$ but the band indices in graphene are taken as the valence band $\nu = \nu' = v$. The remaining integrals can be evaluated within a $\mathbf{k}{\cdot}\mathbf{p}$ expansion. Last we introduce exciton operators in WSe_2 $P^{\mu}_{\mathbf{Q}} = \sum_{\mathbf{q}} \varphi^{\mu}_{\mathbf{q}} c^{\dagger}_{\mathbf{q}+\frac{m_e}{m_h+m_e}\mathbf{Q}} v_{\mathbf{q}-\frac{m_h}{m_h+m_e}\mathbf{Q}}$ with quantum state μ and COM momentum $\mathbf{Q}$. The final Hamiltonian reads

$$H = \sum_{\mathbf{k},\mathbf{Q},\mu} W^{\mu}_{\mathbf{Q}} P^{\dagger\mu}_{\mathbf{Q}} v^{\dagger}_{\mathbf{k}-\mathbf{Q}} v_{\mathbf{k}} + h.c.\,, \tag{8}$$

with the coupling element

$$W_{\mathbf{Q}} = \frac{1}{e} V_{\mathbf{Q}} \mathbf{d}^{cv} \cdot \mathbf{Q} \varphi^{*\mu}(\mathbf{r} = 0). \tag{9}$$

In the following we restrict ourselves to the lowest bound excitons $\mu = 1s$. From this Hamiltonian we calculate the equation of motion for the exciton occupation in the TMD $N_{\mathbf{Q}} = \langle P^{\dagger}_{\mathbf{Q}} P_{\mathbf{Q}} \rangle$ and the electron occupation in the valence band of graphene $f_{\mathbf{k}} = \langle v^{\dagger}_{\mathbf{k}} v_{\mathbf{k}} \rangle$ by exploiting Heisenberg equation of motion.

The resulting equations of motion read

$$\partial_t N_{\mathbf{Q}} = \frac{2\pi}{\hbar} \sum_{\mathbf{k}} |W_{\mathbf{Q}}|^2 \left(f_{\mathbf{k}}(1 - f_{\mathbf{k}-\mathbf{Q}}) - N_{\mathbf{Q}}(f_{\mathbf{k}-\mathbf{Q}} - f_{\mathbf{k}}) \right) \delta(\epsilon_{\mathbf{k}} - \epsilon_{\mathbf{k}-\mathbf{Q}} - E_{\mathbf{Q}}) \tag{10}$$

$$\partial_t f_{\mathbf{k}} = \frac{2\pi}{\hbar} \sum_{\mathbf{Q}} |W_{\mathbf{Q}}|^2 \left(f_{\mathbf{k}-\mathbf{Q}}(1 - f_{\mathbf{k}}) - N_{\mathbf{Q}}(f_{\mathbf{k}} - f_{\mathbf{k}-\mathbf{Q}}) \right) \delta(\epsilon_{\mathbf{k}-\mathbf{Q}} - \epsilon_{\mathbf{k}} - E_{\mathbf{Q}}) \tag{11}$$

$$+ \frac{2\pi}{\hbar} \sum_{\mathbf{Q}} |W_{\mathbf{Q}}|^2 \left(N_{\mathbf{Q}}(f_{\mathbf{k}-\mathbf{Q}} - f_{\mathbf{k}}) - f_{\mathbf{k}}(1 - f_{\mathbf{k}-\mathbf{Q}}) \right) \delta(\epsilon_{\mathbf{k}} - \epsilon_{\mathbf{k}-\mathbf{Q}} - E_{\mathbf{Q}}) \tag{12}$$

Estimation of the decay rate of WSe_2 excitons From the Boltzmann equation we can identify the decay rate of WSe_2 excitons as

$$\Gamma_{\mathbf{Q}} = 8\pi \sum_{\mathbf{k}} (f_{\mathbf{k}-\mathbf{Q}} - f_{\mathbf{k}}) \delta(\epsilon_{\mathbf{k}} - \epsilon_{\mathbf{k}-\mathbf{Q}} - E_{\mathbf{Q}}), \tag{13}$$

where we have added a factor of 4 to account for the valley and spin degree of freedom in graphene. Analyzing the Dirac distribution, we find that $\mathbf{k}$ accounts for electrons close the Dirac point, and $\mathbf{k}-\mathbf{Q}$ for electrons deep in the valence band. In order to get a simple expression for the decay rate, we assume that the electrons close to the Dirac point have much smaller momenta than the electrons deep in the valence band, i .e. $\mathbf{k} \ll \mathbf{Q}$ and $\mathbf{k}+\mathbf{Q} \approx \mathbf{Q}$.

This way, the Dirac distribution and the $\mathbf{k}$ can be evaluated analytically yielding

$$\Gamma_{\mathbf{Q}} = |W_{\mathbf{Q}}|^2 \frac{4}{\hbar v_F} \left(Q - \frac{E_{\mathbf{Q}}}{\hbar v_F}\right) \left(f_{\mathbf{Q}} - f_{Q-\frac{E_{\mathbf{Q}}}{\hbar v_F}}\right) \theta \left(Q - \frac{E_{\mathbf{Q}}}{\hbar v_F}\right) \tag{14}$$

The rate depends on the matrix element of the MA transfer, the density of states in graphene and on the occupation difference of the involved states in graphene which accounts for the Pauli blocking. The heavyside function $\theta\left(Q - \frac{E_{\mathbf{Q}}}{\hbar v_F}\right)$ accounts for the fact, that a minimal momentum is required to fulfil the energy and momentum conservation during the intervalley transfer.

Fig. 4**c** in the main text illustrates the MA rate of WSe_2 excitons to graphene for the photo-induced hole vacancies at different energy of μ_{Gr}^{h*}. We adjusted the graphene dispersion to the results from the ARPES measurement. From $\tau = \hbar/\Gamma$ we find scattering times of 270 fs ($\mu_{Gr}^{h*} = -0.3\,\text{eV}$), 210 fs ($\mu_{Gr}^{h*} = -0.4\,\text{eV}$), and 175 fs ($\mu_{Gr}^{h*} = -0.5\,\text{eV}$).

Origin of the finite center-of-mass (COM) momentum The MA mediated IET requires nonzero COM momentum of the exciton. For example, the required COM momentum is $\sim 1.3\,\text{nm}^{-1}$ as shown in Fig.4**c** (main text) with $\mu_{Gr}^{*} = -0.3$ eV, which corresponds to a kinetic energy of approximately 100 meV based on the effective mass of the exciton $m_{ex} = 0.65 m_e$.[25] Then, where

does the energy (momentum) come from? In the following, we discuss the possible origins of COM momentum of the exciton which is quantified by the kinetic energy assuming the parabolic excitonic band dispersion.

At room temperature, the mean kinetic energy of the excitons is 25.6 meV which is not enough to explain the required energy. Therefore, we calculate the temporal evolution of the exciton energy-momentum occupation during the optical pump for detuned excitation[26]. The equation of motion for the excitonic coherence in the rotating frame reads

$$\dot{P}_{\mathbf{0}}(t) = \frac{1}{i\hbar}\left(E_{\mathbf{0}} - \hbar\omega_L - i\gamma\right) P_{\mathbf{0}}(t) + \mathbf{d}\cdot\mathbf{E}(t), \tag{15}$$

where the first term accounts for the detuning of the excitonic transition energy $E_{\mathbf{0}}$ from the light pulse energy $\hbar\omega_L$. γ accounts for the dephasing of the excitonic coherence with contributions from radiative and exciton phonon coupling[27]. The last term accounts for the optical excitation with the dipole element $\mathbf{d}$ and the exciting electric field $\mathbf{E}(t)$. The equation of motion for the incoherent exciton occupation reads

$$\dot{N}_{\mathbf{Q}} = \Gamma^{Form}_{\mathbf{Q}} |P_{\mathbf{0}}|^2 + \sum_{\mathbf{K}} \Gamma^{in}_{\mathbf{Q},\mathbf{K}} N_{\mathbf{K}} - \sum_{\mathbf{K}} \Gamma^{out}_{\mathbf{Q},\mathbf{K}} N_{\mathbf{Q}}. \tag{16}$$

The first term accounts for the formation of incoherent exciton occupation from phonon induced dephasing from the excitonic coherence. The last two terms account for the thermalization of incoherent excitons[21]. The coupling element of the exciton formation reads

$$\Gamma^{Form}_{\mathbf{Q}} = \frac{2}{\hbar}\sum_{\pm,\alpha} |g_{\mathbf{Q}}|^2 \left(\frac{1}{2} \pm \frac{1}{2} + n^{\alpha}_{\mathbf{Q}}\right) \frac{\gamma}{(E_{\mathbf{Q}} - \hbar\omega_L \mp \hbar\Omega^{\alpha})^2 + \gamma^2}. \tag{17}$$

with the energy $\hbar\Omega^{\alpha}_{\mathbf{Q}}$ and the occupation $n^{\alpha}_{\mathbf{Q}}$ of phonons in the branch α with momentum $\mathbf{Q}$. The $\pm$ summation accounts for phonon emission/absorption processes.

Supplementary Fig. 12**a** illustrates the snapshots of the exciton occupation directly at the maximum of the pump pulse as a function of kinetic energy with selected detuning pump photon energy *above* the excitonic transition energy. The temperatures are set as room temperature for all the calculation. With increasing pump photon energy, the amount of injected excitons decreases due to the non-resonant excitation, eq. 15. However, at larger detunings, excitons occupy larger energy states due to the excess energy of the pump pulse, which is provided by acoustic and optical phonon transitions. The Supplementary Fig. 12**b** illustrates the exciton occupations but normalized to the maximum. Here it is even more obvious that the excitons obtain higher energies as the detuning increases. Interesting, for larger detunings two maxima can be observed, where the higher one originates from the formation of excitons via acoustic phonon scattering. The lower peak originates from the formation of excitons via optical phonon emission but also from relaxation of excitons from the higher peak via optical phonon emission.

Supplementary Fig. 12**c** illustrates snapshots of the exciton occupation directly at the maximum of the pump pulse as a function of kinetic energy for selected detunings *below* the excitonic transition energy. Similar to the previous scenario, the density of injected excitons decreases with increasing detuning due to the non-resonant excitation, eq. 15. In Supplementary Fig. 12**d**, the energy-dependent exciton occupations are normalized to their maximum. Interestingly, for pumping with larger negative detunings, the exciton distribution broadens such that the relative exciton occupation at large kinetic energies increases. The reason is, that for larger detunings the Lorentzian in equation 17 flattens which results in higher occupation of hot exciton at large energy range. As a consequence, for the near-resonant excitation below the excitonic transition,

a substantial amount of excitons is formed at energies above 100 meV which contribute to the Meitner-Auger scattering. To conclude, non-resonant excitation of the exciton, both above and below the resonance, introduce hot excitons with high kinetic energy, which are subjected to the Meitner-Auger IET.

In our experiment, the pump photon energy is $\hbar\nu_{pump} = 1.55$ eV and the A-exciton transition energy is $E_{ex} = 1.63$ eV determined by the energy difference of excited-state particles at CBM and VBM. After the photoexcition which prepares the *coherent* excitons with zero COM momentum (Supplementary Fig. 12**e**), the phonon-assisted dephasing process transfers the coherent excitons to *incoherent* exciton population which gain the finite COM momenta (Supplementary Fig. 12**f**). This dephasing process has been observed by our previous study[28]. The subsequent thermalization of excitons at the excitonic states (Supplementary Fig. 12**g**) also contribute to the nonzero COM momenta which is already included in our calculation.

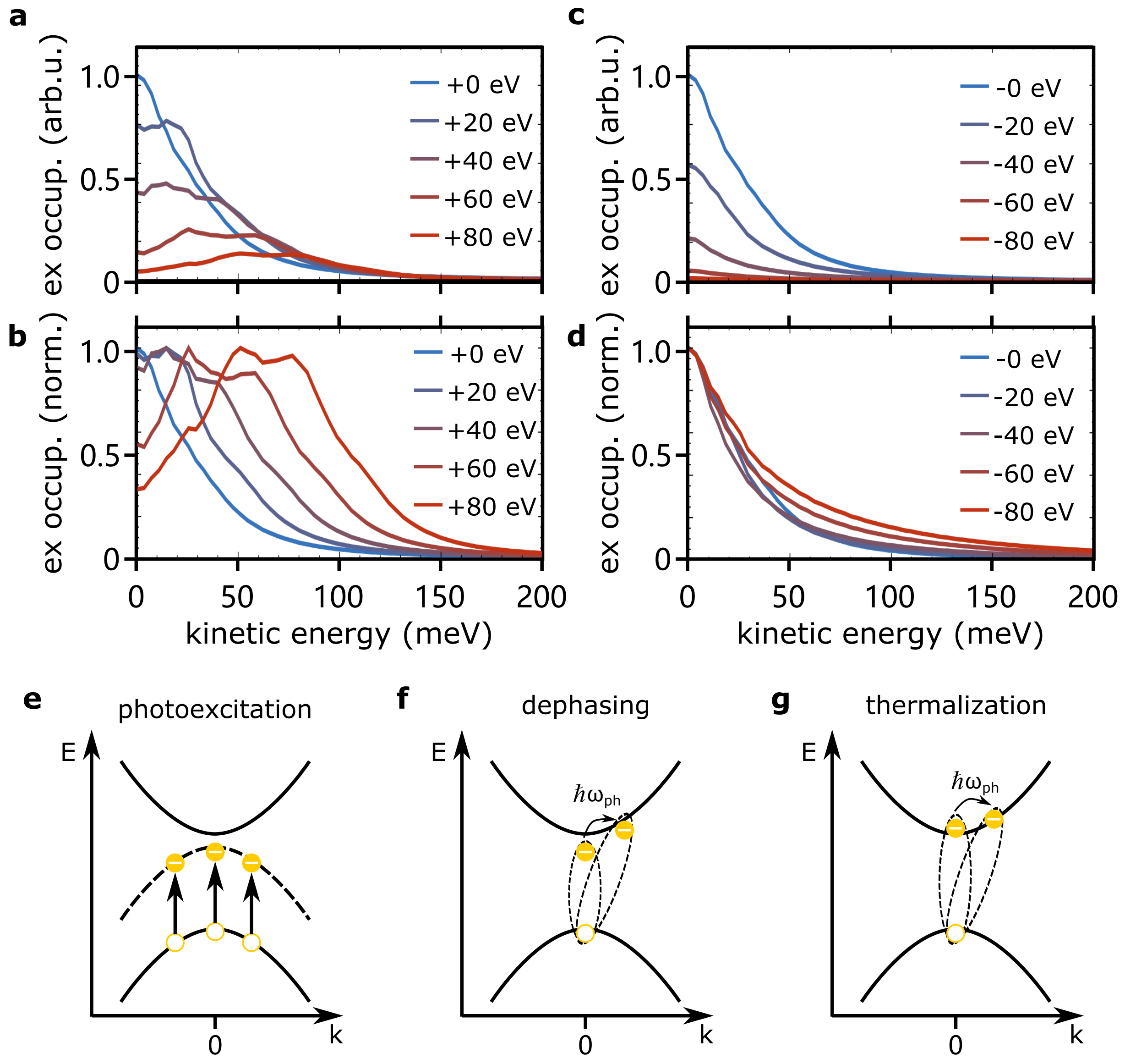

Supplementary Fig. 12: Finite COM momentum of excitons at the $\mathrm{K_{WSe_2}}$ valley. **a**, The calculated energy-dependent hot exciton occupation with detuned pump photon energy above the resonant excitonic transition energy. **b**, Normalized hot exciton distributions in **a**. **c** The hot exciton occupation with detuned pump photon energy below the resonant excitonic transition energy and **d** is the corresponding normalized hot exciton distributions.

Schematic illustrations of involved ultrafast dynamics: **e**, photoexcitation creates the coherent exciton. The dash curve represents the coherent excitonic state. **f**, The phonon-assisted dephasing process transfers coherent excitons to incoherent excitons, at the same time, increases the COM momentum of excitons. **g**, The following thermalization also contributes to the finite COM momentum.

Förster coupling To calculate the Förster rate from WSe_2 to graphene, we start with the Hamiltonian

$$H_F = \sum_{\mathbf{k},\mathbf{q},\mathbf{k}',\mathbf{q}',\lambda,\lambda',\nu,\nu'} V^{\lambda\nu\nu'\lambda'}_{\mathbf{k},\mathbf{q},\mathbf{q}',\mathbf{k}'} \lambda^\dagger_{\mathbf{k}} \nu^\dagger_{\mathbf{q}} \nu'_{\mathbf{q}'} \lambda'_{\mathbf{k}'}. \tag{18}$$

As a convention, we use $\lambda^{(\prime)}$ as band indices and $\mathbf{k}^{(\prime)}$ as momenta in WSe_2 layer and $\nu^{(\prime)}$ as band indices and $\mathbf{q}^{(\prime)}$ as momenta in graphene. The appearing matrix element reads

$$V^{\lambda\nu\nu'\lambda'}_{\mathbf{k},\mathbf{q},\mathbf{q}',\mathbf{k}'} = \int_{\mathbb{R}^3} d^3r \int_{\mathbb{R}^3} d^3r' \Psi^{\lambda *}_{\mathbf{k}}(\mathbf{r}) \Psi^{\nu *}_{\mathbf{q}}(\mathbf{r}') V(\mathbf{r},\mathbf{r}') \Psi^{\nu'}_{\mathbf{q}'}(\mathbf{r}') \Psi^{\lambda'}_{\mathbf{k}'}(\mathbf{r}). \tag{19}$$

Here, $\Psi^{\lambda/\nu}_{\mathbf{k}/\mathbf{q}}$ account for the electronic Bloch waves in WSe_2 and graphene. The appearing Coulomb potential shall take into account the dielectric environment of the heterostructure, including the WSe_2 and graphene layer which are distanced by a gap with dielectric constant ϵ_R and width z (closely stacked structures have $z = 0$)[29]. Additionally we take substrates below and above the structure into account.

We can evaluate the matrix element by Fourier transforming the Coulomb potential and calculating the real space integrals within a $\mathbf{k} \cdot \mathbf{p}$ expansion[30]. We introduce exciton operators in WSe_2 $P^\mu_{\mathbf{Q}} = \sum_{\mathbf{q}} \varphi^\mu_{\mathbf{q}} c^\dagger_{\mathbf{q}+\frac{m_e}{m_h+m_e}\mathbf{Q}} v_{\mathbf{q}-\frac{m_h}{m_h+m_e}\mathbf{Q}}$ with quantum state μ and COM momentum $\mathbf{Q}$ as

well as pair operators in graphene $R^{\mathbf{q}}_{\mathbf{Q}} = c^{\dagger}_{\mathbf{q}+\frac{1}{2}\mathbf{Q}} v_{\mathbf{q}-\frac{1}{2}\mathbf{Q}}$. The Hamiltonian then reads

$$H_F = \sum_{\mathbf{Q},\mathbf{q},\mu} F^{\mu}_{\mathbf{Q}}(z) P^{\dagger\mu}_{\mathbf{Q}} R^{\mathbf{q}}_{\mathbf{Q}} + h.c. \tag{20}$$

The appearing coupling element reads

$$F^{\mu}_{\mathbf{Q}}(z) = \frac{1}{e^2\sqrt{A}} V_{\mathbf{Q}}(z) \varphi^{\mu}(\mathbf{r}=0) \mathbf{Q}\cdot \mathbf{d_T}^{cv} \mathbf{Q}\cdot \mathbf{d}^{vc}_{G} \tag{21}$$

with $\mathbf{d}^{vc}_{T}$ the dipole element in WSe_2, $\mathbf{d}^{vc}_{G}$ the dipole element in graphene, $\varphi^{\mu}(\mathbf{r})$ the excitonic wave function in real space with quantum number μ in WSe_2. We restrict our analysis to the lowest lying excitons $\mu = 1s$.

The Förster induced transition rate is given as [31]

$$\Gamma_{\mathbf{Q}}(z) = 4\pi \sum_{\mathbf{q}} |F_{\mathbf{Q}}(z)|^2 \delta\left(E^{\mathbf{q}}_{\mathbf{Q}} - E^{1s}_{\mathbf{Q}}\right), \tag{22}$$

where we have already added a factor of 2 to account for the electron spin in graphene. We analytically treat the summation over the delta function, where the area which appears in equation (21) cancels. We arrive at

$$\Gamma_{\mathbf{Q}}(z) = \frac{|F_{\mathbf{Q}}(z)|^2 A E^{1s}_{\mathbf{Q}}}{2\hbar^2 v_F^2}. \tag{23}$$

v_F is the Fermi velocity in graphene. The area A cancels with the area in $|F_{\mathbf{Q}}(z)|$. In a last step we average over the angle dependence of $|F_{\mathbf{Q}}(z)|^2$, und sum the result over the K and K' point in graphene, which is already included in the $\mathbf{q}$ summation in eq. 22. This way we arive at the final expression

$$\Gamma_{\mathbf{Q}}(z) = \frac{|V_{\mathbf{Q}}(z)|^2 |\varphi^{1s}(\mathbf{r}=0)| d_T^2 d_G^2 E^{1s}_{\mathbf{Q}} Q^4}{8\hbar^2 v_F^2 e^2}. \tag{24}$$

The Coulomb potential $V_{\mathbf{Q}}(z)$ is given as

$$V_{\mathbf{Q}}(z) = \frac{e^2}{\epsilon_0 |\mathbf{Q}| \epsilon_{\mathbf{Q}}(z)}, \tag{25}$$

where the momentum dependent dielectric function $\epsilon_{\mathbf{Q}}(z)$ accounts for the dielectric screening from the surrounding[29]. As input parameters, we require the thickness of graphene and WSe_2 layers and their respective dielectric constants. Note, that in the limit of infinitely thin films and a uniform background, our results coincides with our previous one[21]. The required parameters are listed in table 1.

Fig. 4**d** in the main text illustrates the Förster transfer rate as a function of COM momentum and for different WSe_2 - graphene distances. For $\mathbf{Q} = 0$ we find a vanishing Förster rate followed by a monotonous increase. The large $\mathbf{Q}$ behavior is dictated by the interplay of the momentum dependence of the Coulomb potential and the factor Q^4. For the closest stacking, i.e. 0.0 nm we find a peak transition rate of about 0.08 meV.

Now, we would like to emphasize the difference between MA and Förster transfer theoretically. The MA and Förster transfer are related and can be derived from the same Hamiltonian since the physical origin of both mechanisms is the Coulomb interaction between the involved two-dimensional materials. However, the MA-type IET can be described as monopole-dipole interaction which is often neglected in the Coulomb potential and different from the dipole-dipole coupling term corresponding to the Förster-type IET. To illustrate this, we present the detailed derivations of Dexter, Förster, and MA transfer using a multipole expansion of the Coulomb interaction. We start from the Coulomb Hamiltonian involving the typical Coulomb matrix element. Here, we use a

simple Coulomb potential $V(\mathbf{r}-\mathbf{r}')=\frac{e_0^2}{4\pi\epsilon_0\epsilon}\frac{1}{|\mathbf{r}-\mathbf{r}'|}$ with a constant uniform background. However, in the manuscript, we use a more complex nonlinear dielectric function valid for the TMDC-graphene interface and treat it in the momentum space. The Hamiltonian involving all transfer processes (Förster, Dexter, MA) reads

$$H=\sum_{\substack{\lambda,\lambda',\nu,\nu'\\ \mathbf{k},\mathbf{k}',\mathbf{q},\mathbf{q}'}} V^{\lambda\nu\nu'\lambda'}_{\mathbf{k},\mathbf{q},\mathbf{q}',\mathbf{k}'}\lambda^{\dagger}_{\mathbf{k}}\nu^{\dagger}_{\mathbf{q}}\nu'_{\mathbf{q}'}\lambda'_{\mathbf{k}'} \tag{26}$$

$$V^{\lambda\nu\nu'\lambda'}_{\mathbf{k},\mathbf{q},\mathbf{q}',\mathbf{k}'}=\int d^3r\int d^3r'\;\Psi^*_{\lambda,\mathbf{k}}(\mathbf{r})\Psi^*_{\nu,\mathbf{q}}(\mathbf{r}')V(\mathbf{r}-\mathbf{r}')\Psi_{\nu',\mathbf{q}'}(\mathbf{r}')\Psi_{\lambda',\mathbf{k}'}(\mathbf{r})\;, \tag{27}$$

where $(\lambda^{(\prime)},\mathbf{k}^{(\prime)})$ stand for graphene quantum numbers and $(\nu^{(\prime)},\mathbf{q}^{(\prime)})$ for WSe_2 quantum numbers. We provide now a detailed derivation of all these processes:

The spatial vector $\mathbf{r}$ can be decomposed into a lattice vector $\mathbf{R}_n$ pointing to the nth unit cell and a vector $\mathbf{r}_n$ defined locally in the nth unit cell: $\mathbf{r}\rightarrow\mathbf{r}_n+\mathbf{R}_n$. Then the spatial integral is changed into a sum over all unit cells and an integration over one unit cell. For electronic Bloch functions of the form $\Psi_{\lambda,\mathbf{k}}=\xi_{\mathbf{k}}(\mathbf{r})u_{\lambda,\mathbf{k}}(\mathbf{r})$, with lattice periodic function $u_{\lambda,\mathbf{k}}(\mathbf{r})$ and envelope $\xi_{\mathbf{k}}(\mathbf{r})$, we obtain

$$\begin{aligned}V^{\lambda\nu\nu'\lambda'}_{\mathbf{k},\mathbf{q},\mathbf{q}',\mathbf{k}'}&=\sum_{\mathbf{R}_n,\mathbf{R}'_n}\xi^*_{\mathbf{k}}(\mathbf{R}_n)\xi^*_{\mathbf{q}}(\mathbf{R}'_n)\xi_{\mathbf{q}'}(\mathbf{R}'_n)\xi_{\mathbf{k}'}(\mathbf{R}_n)\\&\quad\times\int_{UC}d^3r_n\int_{UC}d^3r'_n\;u^*_{\lambda,\mathbf{k}}(\mathbf{r})u^*_{\nu,\mathbf{q}}(\mathbf{r}')V(\mathbf{r}_n-\mathbf{r}'_n+\mathbf{R}_n-\mathbf{R}'_n)u_{\nu',\mathbf{q}'}(\mathbf{r}')u_{\lambda',\mathbf{k}'}(\mathbf{r})\;.\end{aligned} \tag{28}$$

We assumed that the envelope is spatially constant over one unit cell and exploited the periodicity

of the functions $u_{\lambda,\mathbf{k}}(\mathbf{r})$. Next, we Taylor expand the Coulomb potential at two points $(\mathbf{R}_n - \mathbf{R}'_n)$:

$$\begin{aligned}\frac{1}{|\mathbf{r}_n - \mathbf{r}'_n + \mathbf{R}_n - \mathbf{R}'_n|} = & \frac{1}{|\mathbf{R}_n - \mathbf{R}'_n|} \\ & + \frac{(\mathbf{R}_n - \mathbf{R}'_n)}{|\mathbf{R}_n - \mathbf{R}'_n|^3} \cdot (\mathbf{r}'_n - \mathbf{r}_n) \\ & + \frac{\mathbf{r}_n \cdot \mathbf{r}'_n}{|\mathbf{R}_n - \mathbf{R}'_n|^3} - 3\frac{(\mathbf{r}'_n \cdot (\mathbf{R}_n - \mathbf{R}'_n))((\mathbf{R}_n - \mathbf{R}'_n) \cdot \mathbf{r}_n)}{|\mathbf{R}_n - \mathbf{R}'_n|^5} \, . \end{aligned} \tag{29}$$

The first line is the zeroth-order and corresponds to monopole-monopole interaction between the two parts of the heterostructure. The second line is the monopole-dipole interaction which corresponds to an intraband-interband coupling. This term is often neglected in a rotating wave approximation when applied to gaped structures, such as a semiconductor heterostructure. However, in the case of graphene, this term can not be ignored since the vanishing bandgap allows energetically favourite intraband transitions (if not Pauli-blocked). Here, a coupling of intraband excitation in graphene and excitons in TMDCs can occur. A typical effect is carrier multiplication[32,33]. In our study, we include this term which gives rise to the MA coupling, as a monopole (graphene)-dipole (TMDC) interaction. The last line, appearing in the first order of both arguments $\mathbf{r}_n$, $\mathbf{r}'_n$, constitutes the typical dipole-dipole interaction coupling (Förster-type transfer).

Coming back to the Hamiltonian and inserting the last line of Taylor expansion (Eq. (29)) into Eq. (28), we obtain the Förster-type coupling,

$$\begin{aligned} V^{\lambda\nu\bar{\nu}\bar{\lambda}}_{\mathbf{k},\mathbf{q},\mathbf{q}',\mathbf{k}'} = & \frac{1}{4\pi\epsilon_0\epsilon} \sum_{\mathbf{R}_n,\mathbf{R}'_n} \xi^*_{\mathbf{k}}(\mathbf{R}_n)\xi^*_{\mathbf{q}}(\mathbf{R}'_n)\xi_{\mathbf{q}'}(\mathbf{R}'_n)\xi_{\mathbf{k}'}(\mathbf{R}_n) \\ & \times \left(\frac{\mathbf{d}^{\lambda\bar{\lambda}}_{\mathbf{k},\mathbf{k}'} \cdot \mathbf{d}^{\nu\bar{\nu}}_{\mathbf{q},\mathbf{q}'}}{|\mathbf{R}_n - \mathbf{R}'_n|^3} - 3\frac{\mathbf{d}^{\lambda\bar{\lambda}}_{\mathbf{k},\mathbf{k}'} \cdot (\mathbf{R}_n - \mathbf{R}'_n)\mathbf{d}^{\nu\bar{\nu}}_{\mathbf{q},\mathbf{q}'}(\mathbf{R}_n - \mathbf{R}'_n)}{|\mathbf{R}_n - \mathbf{R}'_n|^5} \right) \end{aligned} \tag{30}$$

with the dipole matrix elements of both materials defined as $\mathbf{d}^{\lambda\bar{\lambda}}_{\mathbf{k},\mathbf{k}'} = e_0 \int_{UC} d^3r \; u^*_{\lambda,\mathbf{k}}(\mathbf{r})\mathbf{r}u_{\bar{\lambda},\mathbf{k}'}(\mathbf{r})$

where $\bar{\lambda} \neq \lambda$. As explicitly expressed, the Förster-type transfer is referred to as a dipole-dipole coupling.

Next, we insert the second line of Eq. (29) into Eq. (28) to investigate the MA-type coupling:

$$\begin{aligned} V^{\lambda\nu\nu'\lambda'}_{\mathbf{k},\mathbf{q},\mathbf{q}',\mathbf{k}'} &= \frac{e_0^2}{4\pi\epsilon_0\epsilon} \sum_{\mathbf{R}_n,\mathbf{R}'_n} \xi^*_{\mathbf{k}}(\mathbf{R}_n)\xi^*_{\mathbf{q}}(\mathbf{R}'_n)\xi_{\mathbf{q}'}(\mathbf{R}'_n)\xi_{\mathbf{k}'}(\mathbf{R}_n)\frac{(\mathbf{R}_n - \mathbf{R}'_n)}{|\mathbf{R}_n - \mathbf{R}'_n|^3} \\ &\times \left(\int_{UC} d^3r_n \; u^*_{\lambda,\mathbf{k}}(\mathbf{r}_n)u_{\lambda',\mathbf{k}'}(\mathbf{r}_n) \int_{UC} d^3r'_n \; u^*_{\nu,\mathbf{q}}(\mathbf{r}'_n)\mathbf{r}'_n u_{\nu',\mathbf{q}}(\mathbf{r}'_n) \right. \\ &\left. - \int_{UC} d^3r_n \; u^*_{\lambda,\mathbf{k}}(\mathbf{r}_n)\mathbf{r}_n u_{\lambda',\mathbf{k}'}(\mathbf{r}_n) \int_{UC} d^3r'_n \; u^*_{\nu,\mathbf{q}}(\mathbf{r}'_n)u_{\nu',\mathbf{q}}(\mathbf{r}'_n)\right) \; . \end{aligned} \tag{31}$$

Here, the second line of Eq. (31) describes the interaction of a TMDC interband transition (r'_n-integral, $(\nu, \mathbf{q})$ WSe_2 quantum numbers) and an intraband graphene transition (r_n-integral, $(\lambda, \mathbf{k})$ graphene quantum numbers). Note, the third line of Eq. (31) describes an interband transition in the graphene layer and an intraband transition in the WSe_2 layer. It is not feasible in our experiment and also unlikely from theoretical considerations. Thus, we neglect this term. Finally, we obtain

$$V^{\lambda\nu\bar{\nu}\lambda}_{\mathbf{k},\mathbf{q},\mathbf{q}',\mathbf{k}'} = \frac{e_0}{4\pi\epsilon_0\epsilon} \sum_{\mathbf{R}_n,\mathbf{R}'_n} \xi^*_{\mathbf{k}}(\mathbf{R}_n)\xi^*_{\mathbf{q}}(\mathbf{R}'_n)\xi_{\mathbf{q}'}(\mathbf{R}'_n)\xi_{\mathbf{k}'}(\mathbf{R}_n)\frac{(\mathbf{R}_n - \mathbf{R}'_n)}{|\mathbf{R}_n - \mathbf{R}'_n|^3}\mathbf{d}^{\nu\bar{\nu}}_{\mathbf{q},\mathbf{q}'}\delta_{\mathbf{k},\mathbf{k}'} \; , \tag{32}$$

where we used the orthogonality of the lattice periodic functions to solve the r_n-integral. As explicitly shown in Eq. (32), only one dipole element is involved in MA-type transfer, distinguishable from the Förster-type dipole-dipole coupling.

Distance dependence of Meitner-Auger-like and Förster-type energy transfer To study the distance dependence of the Meitner-Auger transfer and compare it to the known dependence of the Förster process, we calculate MA- and Förster-type transfer rates as a function of interlayer distance z. In Supplementary Fig. 13 we show the z-dependence of the Meitner-Auger transfer

and also the Förster result, from which we observe a very different behaviour at long distances. For the Förster transfer, we obtain an exponential decay ($\exp(-z)$) in the near-field and the well-known z^{-4} dependence in the far-field. In contrast to the Förster transfer, the Meitner-Auger transfer decays solely exponentially. In principle, these two processes could be distinguished by the different behaviours at long distances. However, both processes decay exponentially at short distances, where they can not be distinguished. To formally study the z-dependence of both mechanisms, we assume a thermal distribution of the initial states. The corresponding Q-dependent rate is then averaged over this thermal distribution (cp. for instance, the approach of reference 14 in the main text). To study the behaviour analytically, we assume a uniform dielectric environment.

The detailed computations are shown in the following: The Meitner-Auger rate reads $\Gamma_{\mathbf{Q}} = |W_{\mathbf{Q}}|^2 \frac{4}{\hbar v_F}(Q - E_{\mathbf{Q}}/\hbar v_F)(f_{\mathbf{Q}} - f_{E_{\mathbf{Q}}-Q/\hbar v_F})\theta(Q - E_{\mathbf{Q}}/\hbar v_F)$, where $W_{\mathbf{Q}}$ carries a general Coulomb potential depending on the dielectric environment. In the manuscript we consider a complex non-linear dielectric function to accurately describe the two-dimensional materials on the substrate and evaluate the matrix element in momentum space. However, to have analytical insights, we assume now a Coulomb potential of the form $V(\mathbf{r} - \mathbf{r}') = \frac{1}{4\pi\epsilon\epsilon_0}\frac{1}{|\mathbf{r}-\mathbf{r}'|}$ with uniform background. We can then start from the Coulomb matrix element Eq. (32) forming $W_{\mathbf{Q}}$ in the Meitner-Auger rate. We perform a coordinate transformation with the new coordinates $\mathbf{s} = (\mathbf{R}_n + \mathbf{R}_n' + \mathbf{z})/2$ and $\mathbf{S} = \mathbf{R}_n - \mathbf{R}_{\mathbf{n}}' - \mathbf{z}$. Equation (32) becomes

$$V^{\lambda\nu\bar{\nu}\lambda}_{\mathbf{k},\mathbf{q},\mathbf{q}',\mathbf{k}'} = \frac{1}{4\pi\epsilon_0\epsilon}\sum_{\mathbf{s},\mathbf{Q}} e^{-i\mathbf{Q}\cdot(\mathbf{s}+\mathbf{z})}\frac{\mathbf{s}+\mathbf{z}}{|\mathbf{s}+\mathbf{z}|^3}\delta_{\mathbf{Q},\mathbf{k}-\mathbf{k}'}\delta_{\mathbf{Q},\mathbf{q}-\mathbf{q}'}\mathbf{d}^{\nu\bar{\nu}}_{\mathbf{q},\mathbf{q}-\mathbf{Q}}\,. \quad (33)$$

The dipole element can be written as $\mathbf{d}^{\nu\bar{\nu}}_{\mathbf{q},\mathbf{q}-\mathbf{Q}} = d^{\nu\bar{\nu}}_{\mathbf{q},\mathbf{q}-\mathbf{Q}}\mathbf{e}$ with polarization vector $\mathbf{e}$. The Hamiltonian

Eq. (26) reads

$$H = \sum_{\substack{\lambda,\nu \\ \mathbf{q},\mathbf{Q},\mathbf{k}}} \frac{1}{4\pi\epsilon_0\epsilon} d^{\nu\bar{\nu}}_{\mathbf{q}-\mathbf{Q}/2,\mathbf{q}+\mathbf{Q}/2,} a(\mathbf{Q},\mathbf{z})\ \lambda^{\dagger}_{\mathbf{k}+\mathbf{Q}/2} \nu^{\dagger}_{\mathbf{q}-\mathbf{Q}/2} \bar{\nu}^{\dagger}_{\mathbf{q}+\mathbf{Q}/2} \lambda^{\dagger}_{\mathbf{k}-\mathbf{Q}/2} \tag{34}$$

$$\text{with} \quad a(\mathbf{Q},\mathbf{z}) = \int d^2s\ \frac{e^{-i\mathbf{Q}\cdot(\mathbf{s}+\mathbf{z})}}{|\mathbf{s}+\mathbf{z}|^3} (\mathbf{s}+\mathbf{z})\cdot\mathbf{e}\ . \tag{35}$$

The integral can be solved and reads

$$a(\mathbf{Q},z) = -2\pi i \frac{e^{-Qz}}{Q} \mathbf{Q}\cdot\mathbf{e}\ . \tag{36}$$

To evaluate the z-dependence of the Meitner-Auger rate, we perform a thermal average according to our previous work[31]:

$$\frac{1}{\tau_T} = \langle\frac{1}{\tau}\rangle_T = \frac{1}{\Omega}\int d^2Q\ e^{-\beta E_{\mathbf{Q}}} \frac{1}{\tau_{\mathbf{Q}}} = \frac{2}{\hbar\Omega}\int d^2Q\ e^{-\beta E_{\mathbf{Q}}} \Gamma_{\mathbf{Q}} \tag{37}$$

with $\Omega = \int d^2Q\ \exp(-\beta E_{\mathbf{Q}})$ and $\beta = 1/k_BT$. To calculate the integral, we set the out-scattering and in-scattering occupation $f_{\mathbf{Q}}$ and $f_{E_{\mathbf{Q}}-Q/\hbar v_F}$ to one and zero, which correspond to a fairly rough approximation. The matrix element $|W_{\mathbf{Q}}|^2$ is determined by the function $|a(\mathbf{Q},z)|^2 = 2\pi^2\exp(-2Qz)$ after an angle average. The solution of the integral reads

$$\begin{aligned} \frac{1}{\tau_T} = \frac{\pi^2}{2\hbar\lambda(T)} e^{-\frac{E_{1s}(E_{1s}\lambda^2(T)+2\hbar v_F z)}{\hbar^2 v_F^2}} & \left[-2\hbar\lambda(T)v_F z \right. \\ & \left. + e^{\frac{(E_{1s}\lambda^2(T)+\hbar v_F z)^2}{\hbar^2 v_F^2 \lambda^2(T)}} \sqrt{\pi}\left(2E_{1s}\lambda^2(T)z + \hbar v_F(\lambda^2(T)+2z^2)\right) \mathrm{erfc}\left(\frac{E_{1s}\lambda(T)}{\hbar v_F} + \frac{z}{\lambda(T)}\right)\right]\ , \end{aligned} \tag{38}$$

where we introduced the thermal wavelength $\lambda(T) = \hbar/\sqrt{2Mk_BT}$. In Supplementary Fig. 13 we show the z-dependence of the Meitner-Auger transfer and also the Förster result. In contrast to the Förster tansfer, the Meitner-Auger transfer decays exponentially. From the function $a(Q,z) \propto \exp(-Qz)$ we see that the main contribution stems from momenta Q at $Q \approx 0$. But to account

for energy and momentum conservation, the Meitner-Auger transfer requires large Q, ensured by the Heaviside function in $\Gamma_{\mathbf{Q}}$. We see from the exponential function in front of the parenthesis, that the strength of the exponential decay is determined by the slope of the linear graphene band structure. For the Förster transfer the function $a(Q, z)$ is proportional to $Q \exp(-Qz)$ resulting in the different far-field behavior. Interestingly, the different far-field behavior can be traced back to the linear band structure of graphene, reflected by the Heaviside function in $\Gamma_{\mathbf{Q}}$, and not to the difference in the interaction. When we artificially set the TMDC band gap to zero, the Meitner-Auger transfer shows the same z-dependence as the Förster transfer in the near- and far-field. At last, we want to stress, that to calculate Eq. (38) we assumed thermalized electron occupations in graphene, which is obviously not the case for a transient experiment.

Compared with a heterostructure made by the exfoliation and stacking technique, the epitaxially grown heterostructure provides a shorter interlayer distance, which benefits the interfacial energy transfer. We have identified the closely stacked WSe_2 and graphene layer without any significant spatial gap with atomic force microscopy in a similar heterostructure[6]. The z-dependence of the energy transfer rates is shown in Supplementary Fig.13. It demonstrates a larger transfer energy rate at a shorter z-distance for both MA and Förster-type IET processes. A different IET mechanism could be dominant with increased interlayer distance. However, not only the interlayer distance z determines the interlayer coupling, but also the rotational orientation between two layers. The band structure alignment of an epitaxially grown heterostructure is favoured in a few discrete orientations, for example, twisting angle of $0\,\mathrm{deg}$ and $60\,\mathrm{deg}$ between the WSe_2 and graphene layer. The heterostructures prepared by the exfoliation method provide the flexibility for

engineering the band structure alignment by turning the twisting angel. Therefore, the heterostructure samples prepared by these methods provide their specific way for us to understand the interlayer interaction.

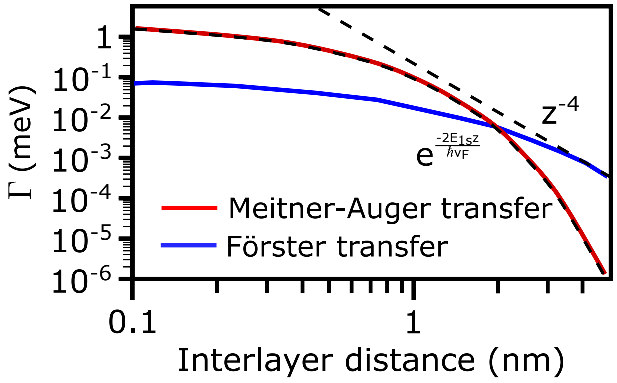

Supplementary Fig. 13: Meitner-Auger and Förster transfer rate as function of layer distance. The Förster transfer rate decays exponentially at short distance and goes over into a z^{-4} dependence (blue). The Meitner-Auger transfer rate continues its exponential decay (red). The strength of the exponential decay of Meitner-Auger rate depends on the slope of the linear graphene band structure

Dexter Coupling The IET process could also mediated by Dexter-type two-particle exchange, whose transfer rate is determined by the wave function overlap[34]. Starting point for the calculation is the Hamiltonian

$$H_D = \sum_{\mathbf{k},\mathbf{q},\mathbf{k}',\mathbf{q}'} V^{cvvc}_{\mathbf{k},\mathbf{q},\mathbf{k}',\mathbf{q}'} c^\dagger_{\mathbf{k}} v^\dagger_{\mathbf{q}} v_{\mathbf{k}'} c_{\mathbf{q}'} + h.c., \tag{39}$$

with the same conventions for the notation as for the calculation of the Förster transfer. The appearing coupling element is defined as

$$V^{cvvc}_{\mathbf{k},\mathbf{q},\mathbf{k}',\mathbf{q}'} = \int_{\mathbb{R}^3} d^3r \int_{\mathbb{R}^3} d^3r' \Psi^{c*}_{\mathbf{k}}(\mathbf{r})\Psi^{v*}_{\mathbf{q}}(\mathbf{r}')V(\mathbf{r},\mathbf{r}')\Psi^{v}_{\mathbf{k}'}(\mathbf{r}')\Psi^{c}_{\mathbf{q}'}(\mathbf{r}). \quad (40)$$

The Coulomb potential is translational invariant in the in-plane direction, i.e. $V(\mathbf{r},\mathbf{r}') = V(\mathbf{r}_\parallel - \mathbf{r}'_\parallel, z, z')$. Fourier transforming the Coulomb potential w.r.t. the in-plane components, writing the electronic wave functions as Bloch waves and decomposing the spatial coordinates into one component inside the unit cell and one which addresses the unit cells $\mathbf{r} \to \mathbf{r} + \mathbf{R}$, yields for the coupling element

$$V^{cvvc}_{\mathbf{k},\mathbf{q},\mathbf{k}',\mathbf{q}'} = \frac{1}{A}\sum_{\mathbf{K}} \int_{uc} dz \int_{uc} dz' \chi^c(z)\chi^v(z')V_{\mathbf{K}}(z,z')\delta_{\mathbf{k},\mathbf{q}'+\mathbf{K}}\delta_{\mathbf{q},\mathbf{k}'-\mathbf{K}}, \quad (41)$$

with

$$\chi^\lambda(z) = \frac{1}{V_{uc}} \int_{uc} d^2r_\parallel u^{*\lambda}_{TMD}(\mathbf{r}_\parallel, z) u^\lambda_{Graphene}(\mathbf{r}_\parallel, z) \quad (42)$$

To evaluate the coupling element further, we restrict ourselves to the case with vanishing interlayer spacing. We decompose the z and z' integration into two integrals over WSe_2 and graphene

$$
\begin{aligned}
V^{cvvc}_{\mathbf{k},\mathbf{q},\mathbf{k}',\mathbf{q}'} &= \frac{1}{A}\sum_{\mathbf{K}} \delta_{\mathbf{k},\mathbf{q}'+\mathbf{K}}\delta_{\mathbf{q},\mathbf{k}'-\mathbf{K}}\times \\
&\Big(\int_{WSe_2} dz \int_{WSe_2} dz'\chi^c(z)\chi^v(z')V_{\mathbf{K}}(z,z') \\
&+\int_{WSe_2} dz \int_{Graphene} dz'\chi^c(z)\chi^v(z')V_{\mathbf{K}}(z,z') \\
&+\int_{Graphene} dz \int_{WSe_2} dz'\chi^c(z)\chi^v(z')V_{\mathbf{K}}(z,z') \\
&+\int_{Graphene} dz \int_{Graphene} dz'\chi^c(z)\chi^v(z')V_{\mathbf{K}}(z,z')\Big)
\end{aligned} \tag{43}
$$

Given that the Coulomb potential varies only weakly with each layer, we can replace the z/z' dependence by the position of the layer $z = z_{WSe_2}, z_{Graphene}$ in the Coulomb potential. This way we arrive at

$$
\begin{aligned}
V^{cvvc}_{\mathbf{k},\mathbf{q},\mathbf{k}',\mathbf{q}'} &= \frac{1}{A}\sum_{\mathbf{K}} \delta_{\mathbf{k},\mathbf{q}'+\mathbf{K}}\delta_{\mathbf{q},\mathbf{k}'-\mathbf{K}}\times \\
&\big(\chi^c_{WSe_2}\chi^v_{WSe_2}V_{\mathbf{K}}(z = z_{WSe_2}, z' = z_{WSe_2}) \\
&+\chi^c_{WSe_2}\chi^v_{Graphene}V_{\mathbf{K}}(z = z_{WSe_2}, z' = z_{Graphene}) \\
&+\chi^c_{Graphene}\chi^v_{WSe_2}V_{\mathbf{K}}(z = z_{Graphene}, z' = z_{WSe_2}) \\
&+\chi^c_{Graphene}\chi^v_{Graphene}V_{\mathbf{K}}(z = z_{Graphene}, z' = z_{Graphene})\big)\,,
\end{aligned} \tag{44}
$$

with

$$
\chi^{\lambda}_{WSe_2/Graphene} = \int_{WSe_2/Graphene} dz\chi^{\lambda}(z), \tag{45}
$$

i.e. the contribution of the wave function overlap of the band λ in the individual layers. Assuming, that the integration in both layers contributes equally to the wave function overlap between WSe_2

and graphene in conduction and valence band, i.e. $\chi^{\lambda}_{WSe_2} = \chi^{\lambda}_{Graphene} = \frac{1}{2}\chi^{\lambda}$, we obtain the final expression for the matrix element

$$V^{cvvc}_{\mathbf{k},\mathbf{q},\mathbf{k}',\mathbf{q}'} = \frac{1}{4A}\chi^c\chi^v \sum_{\mathbf{K}} \delta_{\mathbf{k},\mathbf{q}'+\mathbf{K}}\delta_{\mathbf{q},\mathbf{k}'-\mathbf{K}} V^{Dex}_{\mathbf{K}} \tag{46}$$

with

$$\begin{aligned} V^{Dex}_{\mathbf{K}} &= (V_{\mathbf{K}}(z = z_{WSe_2}, z' = z_{WSe_2}) + V_{\mathbf{K}}(z = z_{WSe_2}, z' = z_{Graphene}) \\ &+ V_{\mathbf{K}}(z = z_{Graphene}, z' = z_{WSe_2}) + V_{\mathbf{K}}(z = z_{Graphene}, z' = z_{Graphene}))\,. \end{aligned} \tag{47}$$

Reinserting the result back into the Hamiltonian (and indexing the operators according to the layer, since due to the momentum conservation our convention breaks down) yields

$$H_D = \sum_{\mathbf{K},\mathbf{k},\mathbf{q}} \frac{1}{4A}\chi^c\chi^v V^{Dex}_{\mathbf{K}+\mathbf{k}-\mathbf{q}} c^{\dagger WSe_2}_{\mathbf{k}+\mathbf{K}} v^{\dagger Gr}_{\mathbf{q}-\mathbf{K}} v^{WSe_2}_{\mathbf{k}} c^{Gr}_{\mathbf{q}} + h.c.. \tag{48}$$

So far, the momenta are defined w.r.t. the Γ point in graphene and WSe_2. Redefining the coordinates $\mathbf{k} \to K^W + \mathbf{k}$ and $\mathbf{q} \to K^G + \mathbf{q}$ expresses them w.r.t. the K point in WSe_2/graphene. A projection on excitonic wave functions in WSe_2 yields

$$H_D = -\sum_{\mathbf{K},\mathbf{q},\nu} \left(\frac{1}{4\sqrt{A}}\chi^c\chi^v \sum_{\mathbf{k}} \varphi^{*\nu}_{K^W+\mathbf{k}} V^{Dex}_{K^W-K^G+\mathbf{K}+\mathbf{k}-\mathbf{q}} \right) P^{\dagger\lambda}_{\mathbf{K}} R^{K^G+\mathbf{q}}_{\mathbf{K}} + h.c.. \tag{49}$$

In the Dexter coupling element, the momentum distance between the K points in graphene and the TMD directly enters. We have $|K^G - K^W| \approx 3.6\,\mathrm{nm}^{-1}$. As a first approximation we can

ignore the COM and relative momenta inside the Coulomb potential $V^{Dex}_{K^W-K^G+\mathbf{K}+\mathbf{k}-\mathbf{q}} \approx V^{Dex}_{K^W-K^G}$ and get a first estimate for the Dexter coupling

$$H_D = -\sum_{\mathbf{K},\mathbf{q},\nu} D_{\mathbf{K}} P^{\dagger\nu}_{\mathbf{K}} R^{K^G+\mathbf{q}}_{\mathbf{K}} + h.c., \tag{50}$$

with

$$D_{\mathbf{K}} = \frac{1}{4\sqrt{A}} \chi^c \chi^v \varphi^{*\nu}(\mathbf{r}=\mathbf{0}) V^{Dex}_{K^W-K^G}. \tag{51}$$

Similar to the Förster transfer, we can evaluate the Dexter induced scattering rate for WSe_2 excitons to graphene

$$\Gamma_{\mathbf{Q}} = 2\pi \sum_{\mathbf{q}} |D_{\mathbf{Q}}|^2 \delta(E^{\mathbf{q}}_{\mathbf{Q}} - E^{1s}_{\mathbf{Q}}) \tag{52}$$

with yields

$$\Gamma_{\mathbf{Q}} = \frac{|D_{\mathbf{Q}}|^2 A E_{\mathbf{Q}}}{4\hbar^2 v_F^2}. \tag{53}$$

Supplementary Fig. 14**a** illustrates the Dexter transfer rate from WSe_2 to graphene as a function of the wave function overlap for the same structure as considered for the Förster transfer. Assuming an overlap between the TMD and graphene wave functions of χ =0.039 (see estimation below, Supplementary Fig. 15**a**), we arrive at a Dexter rate of $1.0 \cdot 10^{-6}$meV. This number is small due to the mismatch of the K points of WSe_2 and graphene and due to the small overlap of the wave functions which enters with the fourth power. As long as the COM momentum $\mathbf{Q}$ is much smaller compared to the distance between the K points, the Dexter rate is independent of the COM momentum.

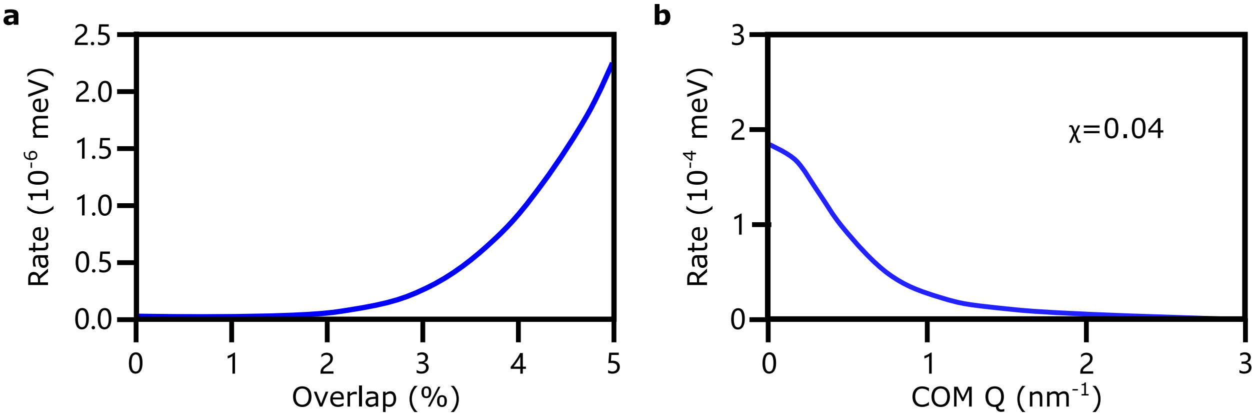

Supplementary Fig. 14: Estimation for the Dexter transfer rate. a, Dexter transfer rate from WSe_2 to graphene as a function of the overlap of the electronic wave functions of graphene and WSe_2. **b**, Dexter transfer rate from WSe_2 to graphene as a function of the excitonic COM momentum $\mathbf{Q}$, with the $\mathrm{K}_{\mathrm{WSe}_2}$ valley and graphene being shifted on top of each others.

Maximum estimation for the Dexter process The reason for the very weak Dexter process is the large momentum mismatch between the K points in WSe_2 and graphene. This generates a momentum-bottleneck. To get an estimation of the maximally possible Dexter rate (and Dexter-like processes, such as Dexter-two-phonon processes), we remove this bottleneck artificially and move the K points of both layers on top of each other. The Hamiltonian reads

$$H_D = -\sum_{\mathbf{K},\mathbf{q},\nu} D_{\mathbf{q},\mathbf{K}} P^{\dagger\lambda}_{\mathbf{K}} R^{\mathbf{q}}_{\mathbf{K}} + h.c.. \tag{54}$$

with the coupling element

$$D_{\mathbf{q},\mathbf{K}} = \frac{1}{4\sqrt{A}} \chi^c \chi^v \sum_{\mathbf{k}} \varphi^{*\nu}_{\mathbf{k}} V^{Dex}_{\mathbf{K}+\mathbf{k}-\mathbf{q}}. \tag{55}$$

The relaxation rate of excitons to graphene is then given by

$$\Gamma_{\mathbf{K}} = \frac{A}{2\pi\hbar}\int_0^{2\pi} d\phi \frac{E_{\mathbf{K}}}{\hbar^2 v_F^2} |D_{\frac{E_{\mathbf{K}}}{\hbar v_F}(cos\phi, sin\phi)^T, \mathbf{K}}|^2. \tag{56}$$

Supplementary Fig. 14**b** illustrates the maximum estimation of the Dexter rate as the function of the COM momentum. We find a relatively weak rate in the order of 10^{-3} meV due to the poor wave function overlap. If we would set $\chi = 1$, we would obtain 70 meV at the maximum. Last, we calculate the thermal average of the Dexter rate, i.e. integrate the momentum dependent Dexter rate together with a normalized Boltzmann distribution at 300 K. We obtain $1.5 \cdot 10^{-4}$ meV.

We would like to note the computation of orbital overlaps is very difficult. Practically, we do not calculate the overlap explicitly but compute the Dexter rate as function of wavefunction overlap. Note, the overlap is not a "totally" unknown parameter in our sample, since we can use the trARPES result as an input. With the below-bandgap excitation (outside the range of the MA transition and therefore constituting as an independent result), we observe an interlayer electron scattering from graphene to WSe_2. By comparing with our calculation of the phonon-assisted charge tunneling rate as function of wavefunction overlap, we could extract an overlap value of around 4%. It can be used to calculate the Dexter-type energy transfer rate in a reasonable range. This wavefunction overlap value is comparable to the *ab-initio* calculated value of the neighboured atoms overlap in graphene, 7%[35], and the interlayer overlap in a $MoSe_2$/WSe_2 heterostructure, 1%[29].

Besides the wavefunction overlap, we present the details of the Dexter transfer calculation

and the applied approximations in the following. We start from the general Coulomb Hamiltonian involving all transfer processes (Förster, Dexter, Meiner-Auger)

$$H = \sum_{\substack{\lambda_1,\lambda_2,\lambda_3,\lambda_4\\ \mathbf{k}_1,\mathbf{k}_2,\mathbf{k}_3,\mathbf{k}_4\\ l_1,l_2,l_3,l_4}} V^{\lambda_1\nu_2\lambda_3\lambda_4 l_1 l_2 l_3 l_4}_{\mathbf{k}_1,\mathbf{k}_2,\mathbf{k}_3,\mathbf{k}_4} \lambda^{\dagger l_1}_{1,\mathbf{k}_1}\lambda^{\dagger l_2}_{2,\mathbf{k}_2}\lambda^{l_3}_{3,\mathbf{k}_3}\lambda^{l_4}_{4,\mathbf{k}_4} \tag{57}$$

$$V^{\lambda_1\nu_2\lambda_3\lambda_4 l_1 l_2 l_3 l_4}_{\mathbf{k}_1,\mathbf{k}_2,\mathbf{k}_3,\mathbf{k}_4} = \int d^3r \int d^3r'\ \Psi^{*l_1}_{\lambda_1,\mathbf{k}_1}(\mathbf{r})\Psi^{*l_2}_{\lambda_2,\mathbf{k}_2}(\mathbf{r}')V(\mathbf{r}-\mathbf{r}')\Psi^{l_3}_{\lambda_3,\mathbf{k}_3}(\mathbf{r}')\Psi^{l_4}_{\lambda_4,\mathbf{k}_4}(\mathbf{r}) \tag{58}$$

with band λ_i, wave vector $\mathbf{k}_i$ and layer l_i. In Eq. (57) we can perform the sum over the band and layer indices. The combination $l_1 = l_3$ and $l_2 = l_4$ and $\lambda_1 = \lambda_4 = c$ and $\lambda_2 = \lambda_3 = v$ corresponds to the Dexter Hamiltonian:

$$H = \sum_{\mathbf{k}_1,\mathbf{k}_2,\mathbf{k}_3,\mathbf{k}_4} V^{cvvcGWGW}_{\mathbf{k}_1,\mathbf{k}_2,\mathbf{k}_3,\mathbf{k}_4} c^{\dagger G}_{\mathbf{k}_1} v^{\dagger W}_{\mathbf{k}_2} v^{G}_{\mathbf{k}_3} c^{W}_{\mathbf{k}_4} + \text{H.c.} = \sum_{\mathbf{k},\mathbf{q},\mathbf{k}',\mathbf{q}'} V^{cvvc}_{\mathbf{k},\mathbf{q},\mathbf{k}',\mathbf{q}'} c^{\dagger}_{\mathbf{k}} v^{\dagger}_{\mathbf{q}} v_{\mathbf{k}'} c_{\mathbf{q}'} + \text{H.c.} \tag{59}$$

where in the second step, we define $\mathbf{k}^{(\prime)}$ as wave vectors in graphene and $\mathbf{q}^{(\prime)}$ as wave vectors in WSe_2, such that we can drop the layer index for graphene (G) and WSe_2 (W). The Hamiltonian Eq. (59) describes the electron transfer from the conduction band of WSe_2 to graphene, together with the valence electron transfer from graphene to WSe_2. The matrix element reads

$$V^{cvvc}_{\mathbf{k},\mathbf{q},\mathbf{k}',\mathbf{q}'} = \frac{1}{A}\sum_{\mathbf{K}} \int d^3r \int d^3r'\ \Psi^{*}_{c,\mathbf{k}}(\mathbf{r})\Psi^{*}_{v,\mathbf{q}}(\mathbf{r}')V_{\mathbf{K}}(z,z')\Psi_{v,\mathbf{q}'}(\mathbf{r}')\Psi_{c,\mathbf{k}'}(\mathbf{r})\ , \tag{60}$$

where we denote the in-plane Fourier transformed Coulomb potential by $V_{\mathbf{K}}(z,z')$.

Now, we calculate the Dexter transfer rate. First, we set the interlayer distance to zero. This yields an upper limit of the Dexter rate in the heterostructure using the proper Coulomb potential. From Eq. (60) we perform the standard steps of shifting the integrals into the first unit

cell $\mathbf{r}_{\parallel}^{(\prime)} \to \mathbf{r}_{\parallel,n}^{(\prime)} + \mathbf{R}_n^{(\prime)}$ and summing over all unit cells $\sum_{n,n'}$. After inserting Bloch functions we obtain

$$V^{cvvc}_{\mathbf{k},\mathbf{q},\mathbf{k}',\mathbf{q}'} = \frac{1}{A}\sum_{\mathbf{K}}\int_{UC} dz \int_{UC} dz' \chi^c(z)\chi^v(z') V_{\mathbf{K}}(z,z')\delta_{\mathbf{k},\mathbf{q}'+\mathbf{K}}\delta_{\mathbf{q},\mathbf{k}'-\mathbf{K}} \ , \tag{61}$$

where the Kronecker delta is obtained from the Bloch wave envelopes. In Eq. (61) we define the orbital overlap $\chi^\lambda(z) = \frac{1}{V_{uc}}\int_{UC} d^2r_\parallel u^{*\lambda}_W(\mathbf{r}_\parallel, z) u^\lambda_G(\mathbf{r}_\parallel, z)$ of WSe_2 (W) and graphene (G) wavefunction. The $z^{(\prime)}$-integration runs over the two materials, such that Eq. (61) can also be written as

$$V^{cvvc}_{\mathbf{k},\mathbf{q},\mathbf{k}',\mathbf{q}'} = \frac{1}{A}\sum_{\mathbf{K}}\delta_{\mathbf{k},\mathbf{q}'+\mathbf{K}}\delta_{\mathbf{q},\mathbf{k}'-\mathbf{K}}\Bigg(\int_W dz\int_W dz'\chi^c(z)\chi^v(z')V_{\mathbf{K}}(z,z') + \int_W dz\int_G dz'\chi^c(z)\chi^v(z')V_{\mathbf{K}}(z,z')$$
$$+\int_G dz\int_W dz'\chi^c(z)\chi^v(z')V_{\mathbf{K}}(z,z') + \int_G dz\int_G dz'\chi^c(z)\chi^v(z')V_{\mathbf{K}}(z,z')\Bigg) \ . \tag{62}$$

To treat the z-integrations in Eq. (62), we assume that the Coulomb potential varies only weakly in the out-of-plane direction (due to the atomic thickness of the two materials). The approach presented here is also performed for the Rytova-Keldysh potential in 2D materials[36–38] and serves as a standard assumption[39–41]. Then, we can use the Coulomb potential directly at the material position and take it out of the integral. We obtain Eq. (62)

$$V^{cvvc}_{\mathbf{k},\mathbf{q},\mathbf{k}',\mathbf{q}'} = \frac{1}{A}\sum_{\mathbf{K}}\delta_{\mathbf{k},\mathbf{q}'+\mathbf{K}}\delta_{\mathbf{q},\mathbf{k}'-\mathbf{K}}\left(\chi^c_W\chi^v_W V_{\mathbf{K}}(z=z_W, z'=z_W)\right.$$
$$\left.+\chi^c_W\chi^v_G V_{\mathbf{K}}(z=z_W,z'=z_G) + \chi^c_G\chi^v_W V_{\mathbf{K}}(z=z_G,z'=z_W) + \chi^c_G\chi^v_G V_{\mathbf{K}}(z=z_G,z'=z_G)\right) \tag{63}$$

with $\chi^\lambda_{W/G} = \int_{W/G} dz\chi^\lambda(z)$ describing the wavefunction overlap of the bands λ of the individual layers (since $\chi^\lambda = \int_{UC} d^2r_\parallel u^*_W(\mathbf{r}_\parallel, z)u_G(\mathbf{r}_\parallel, z)$). Next, we assume that both layers contribute

equally to the wavefunction overlap. This has the consequence that the interlayer and intralayer Coulomb potential contribute equally to the Dexter rate. In an extreme scenario, we calculate the Dexter-type transfer rate with the wavefunction overlap occurring only in one material. It means two times of the intralayer potential will appear instead of the interlayer potential. The Dexter-type transfer rate only increases by a factor of $\sim$1.5, indicating that the transfer process depends mainly on the wavefunction overlap value (which enters to the power of 4 in the Dexter rate) but not where the overlap occurs. By using $\chi_W^\lambda = \chi_G^\lambda = \frac{1}{2}\chi^\lambda$ we obtain the final expression

$$V^{cvvc}_{\mathbf{k},\mathbf{q},\mathbf{k}',\mathbf{q}'} = \frac{1}{4A}\chi^c\chi^v \sum_{\mathbf{K}} \delta_{\mathbf{k},\mathbf{q}'+\mathbf{K}}\delta_{\mathbf{q},\mathbf{k}'-\mathbf{K}} V^{Dex}_{\mathbf{K}} \tag{64}$$

$$V^{Dex}_{\mathbf{K}} = V_{\mathbf{K}}(z = z_W, z' = z_W) + V_{\mathbf{K}}(z = z_W, z' = z_G) + V_{\mathbf{K}}(z = z_G, z' = z_W) + V_{\mathbf{K}}(z = z_G, z' = z_G)\ . \tag{65}$$

When we reinsert the matrix element into the Hamiltonian Eq. (59) and shift the origin of the wave vector on the K points of the respective materials, i.e. $\mathbf{k} \to \mathbf{K}^W + \mathbf{k}$ and $\mathbf{q} \to \mathbf{K}^G + \mathbf{q}$ we obtain

$$H_D = \sum_{\mathbf{K},\mathbf{k},\mathbf{q}} \frac{1}{4A}\chi^c\chi^v V^{Dex}_{\mathbf{K}^W-\mathbf{K}^G+\mathbf{K}+\mathbf{k}-\mathbf{q}} c^{\dagger W}_{\mathbf{K}^W+\mathbf{k}+\mathbf{K}} v^{\dagger G}_{\mathbf{K}^G+\mathbf{q}-\mathbf{K}} v^{W}_{\mathbf{K}^W+\mathbf{k}} c^{G}_{\mathbf{K}^G+\mathbf{q}} + \text{H.c.} \tag{66}$$

Finally, we find that the Fourier transformed Coulomb potential $V^{Dex}_{\mathbf{K}^W-\mathbf{K}^G+\mathbf{K}+\mathbf{k}-\mathbf{q}}$ depends on the momentum difference $\mathbf{K}_W - \mathbf{K}_G + \mathbf{K} + \mathbf{k} - \mathbf{q}$, where $\mathbf{K}_W$ and $\mathbf{K}_G$ are the K points of WSe_2 and graphene respectively, $\mathbf{k}$ and $\mathbf{q}$ are the electronic momenta in WSe_2 and graphene, respectively and $\mathbf{K}$ is the Coulomb induced momentum transfer. From $V^{Dex}_{\mathbf{K}^W-\mathbf{K}^G+\mathbf{K}+\mathbf{k}-\mathbf{q}}$ we see that if a large momentum mismatch between the valleys occurs, the Dexter contribution is reduced. To obtain an upper limit estimate, we approximate $\mathbf{K}_W - \mathbf{K}_G + \mathbf{K} + \mathbf{k} - \mathbf{q} \approx \mathbf{K}_W - \mathbf{K}_G$, to be the dominant distance.

The computation of the Dexter rate is based on the above approximations and the trARPES result of ICT.

Microscopic calculation of interlayer phonon-assisted tunneling process

In this section we will derive an expression for the phonon-assisted tunneling of carriers between the layers. The Hamiltonian of phonon scattering and tunneling can be generally written as

$$H = H_0 + H_1, \tag{67}$$

with H_0 accounting for the dispersion of electrons and phonons.

$$H_0 = \sum_a \epsilon^a a_a^\dagger a_a + \sum_b \hbar\omega^b b_b^\dagger b_b. \tag{68}$$

The first term accounts for the dispersion of carriers with operators $a_a^{(\dagger)}$ and the second term accounts for the dispersion of phonons with operators $b_b^{(\dagger)}$. The quantum numbers a, b account for layer and momentum of the carriers. The interaction Hamiltonian H_1 reads

$$H_1 = \sum_{ab} t^{ab} a_a^\dagger a_b + \sum_{abc} g^{abc} a_a^\dagger a_b (b_c + b_{-c}^\dagger), \tag{69}$$

where the first term represents the tunneling and the second term the scattering of carriers with phonons. Here the notation $-c$ implies, that the momentum has to be inverted, but all other quantum numbers stay the same.

While we are interested in the second order processes of phonon-assisted tunneling, we apply

a canocical transformation to the Hamiltonian

$$H' = e^{-S} H e^{S} = H_0 + \underbrace{(H_1 + [H_0, S])}_{\text{first order}} + \underbrace{\frac{1}{2}[H_1, S]}_{\text{second order}}, \tag{70}$$

and claim that the first order in the interaction vanishes. This holds true for the choice

$$S = \sum_{ab} \alpha_{ab} t^{ab} a^{\dagger} a_b + \sum_{abc} g^{abc} a_a^{\dagger} a_b (\beta_{abc} b_c + \gamma_{abc} b_{-c}^{\dagger}), \tag{71}$$

with coefficients

$$\alpha_{ab} = \frac{1}{\epsilon^b - \epsilon^a}, \tag{72}$$

$$\beta_{abc} = \frac{1}{\epsilon^b - \epsilon^a + \hbar\omega^c}, \tag{73}$$

$$\gamma_{abc} = \frac{1}{\epsilon^b - \epsilon^a - \hbar\omega^c}. \tag{74}$$

The second order Hamiltonian is now given as

$$H_2 = \frac{1}{2}[H_1, S]. \tag{75}$$

Restricting ourselves only to the tunneling-phonon contribution (besides this, also higher order tunneling terms, two-phonon processes as well as attractive electron-electron interaction through phonon interaction are contained in this Hamiltonian) we obtain

$$\begin{aligned} H = & \frac{1}{2} \sum_{abcd} t^{db} g^{adc} a_a^{\dagger} a_b \Big(\big(\frac{1}{\epsilon^b - \epsilon^d} - \frac{1}{\epsilon^d - \epsilon^a + \hbar\omega^c} \big) b_c + \big(\frac{1}{\epsilon^b - \epsilon^d} - \frac{1}{\epsilon^d - \epsilon^a - \hbar\omega^{-c}} \big) b_{-c}^{\dagger} \Big) \\ & - \frac{1}{2} \sum_{abcd} t^{ad} g^{dbc} a_a^{\dagger} a_b \Big(\big(\frac{1}{\epsilon^d - \epsilon^a} - \frac{1}{\epsilon^b - \epsilon^d + \hbar\omega^c} \big) b_c + \big(\frac{1}{\epsilon^d - \epsilon^a} - \frac{1}{\epsilon^b - \epsilon^d - \hbar\omega^{-c}} \big) b_{-c}^{\dagger} \Big) \end{aligned} \tag{76}$$

Now we insert the compounds: $a = (\mathbf{k}_a, \lambda_a, l_a)$ for electrons, where $\mathbf{k}_a$ accounts for the momentum, λ_a accounts for the band and l_a accounts for the layer quantum number. For phonons we insert the compounds $c = (\mathbf{k}_c, l_c, \xi_c)$, with momentum $\mathbf{k}_c$, layer l_c and branch ξ_c and apply the selection rules from the matrix elements:

$$t^{\lambda_b \lambda_d l_b l_d}_{\mathbf{k}_b \mathbf{k}_d} = t^{\lambda_b \lambda_d l_b l_d} \delta^{l_b \bar{l}_d}_{\mathbf{k}_b \mathbf{k}_d} \delta^{\lambda_b \lambda_d}, \tag{77}$$

i.e. the tunneling conserves momentum, and band but changes the layer index, and

$$g^{\lambda_a \lambda_d l_a l_d l_c}_{\mathbf{k}_a \mathbf{k}_d \mathbf{k}_c} = g^{\lambda_a \lambda_d l_a l_d l_c}_{\mathbf{k}_c} \delta^{l_a l_d} \delta^{l_d l_c}_{\mathbf{k}_c, \mathbf{k}_a - \mathbf{k}_d} \delta^{\lambda_a \lambda_d}. \tag{78}$$

i.e. phonon scattering conserves the layer index, but changes the momentum of the carriers.

As a result we obtain

$$\begin{aligned} H = &\frac{1}{2} \sum_{\mathbf{k}\mathbf{K}\lambda l \xi} \lambda^{\dagger \bar{l}}_{\mathbf{k}+\mathbf{K}} \lambda^{l}_{\mathbf{k}} \left(\underbrace{t^{\lambda l \bar{l}} g^{\lambda \bar{l} \xi}_{\mathbf{K}} (\alpha^{\lambda l \bar{l}}_{\mathbf{k}} + \gamma^{\lambda \bar{l} \xi}_{\mathbf{k},\mathbf{K}})}_{s^{\lambda l \bar{l} \xi}_{\mathbf{k},\mathbf{K}}} b^{\bar{l}\xi}_{\mathbf{K}} + \underbrace{t^{\lambda l \bar{l}} g^{\lambda \bar{l} \xi}_{\mathbf{K}} (\alpha^{\lambda l \bar{l}}_{\mathbf{k}} + \beta^{\lambda \bar{l} \xi}_{\mathbf{k},\mathbf{K}})}_{\tilde{s}^{\lambda l \bar{l} \xi}_{\mathbf{k},\mathbf{K}}} b^{\dagger \bar{l} \xi}_{-\mathbf{K}} \right) \\ &- \frac{1}{2} \sum_{\mathbf{k}\mathbf{K}\lambda l \xi} \lambda^{\dagger \bar{l}}_{\mathbf{k}+\mathbf{K}} \lambda^{l}_{\mathbf{k}} \left(\underbrace{t^{\lambda \bar{l} l} g^{\lambda l \xi}_{\mathbf{K}} (\alpha^{\lambda l \bar{l}}_{\mathbf{k}+\mathbf{K}} + \gamma^{\lambda l \xi}_{\mathbf{k},\mathbf{K}})}_{u^{\lambda l \bar{l} \xi}_{\mathbf{k},\mathbf{K}}} b^{l\xi}_{\mathbf{K}} + \underbrace{t^{\lambda \bar{l} l} g^{\lambda l \xi}_{\mathbf{K}} (\alpha^{\lambda l \bar{l}}_{\mathbf{k}+\mathbf{K}} + \beta^{\lambda l \xi}_{\mathbf{k},\mathbf{K}})}_{\tilde{u}^{\lambda l \bar{l} \xi}_{\mathbf{k},\mathbf{K}}} b^{\dagger l \xi}_{-\mathbf{K}} \right), \end{aligned} \tag{79}$$

with

$$\alpha_{\mathbf{k}}^{\lambda ij} = \frac{1}{\epsilon_{\mathbf{k}}^{\lambda i} - \epsilon_{\mathbf{k}}^{\lambda j}} \tag{80}$$

$$\beta_{\mathbf{k},\mathbf{K}}^{\lambda i\xi} = \frac{1}{\epsilon_{\mathbf{k}+\mathbf{K}}^{\lambda i} - \epsilon_{\mathbf{k}}^{\lambda i} + \hbar\omega_{-\mathbf{K}}^{i\xi}} \tag{81}$$

$$\gamma_{\mathbf{k},\mathbf{K}}^{\lambda i\xi} = \frac{1}{\epsilon_{\mathbf{k}+\mathbf{K}}^{\lambda i} - \epsilon_{\mathbf{k}}^{\lambda i} - \hbar\omega_{\mathbf{K}}^{i\xi}} \tag{82}$$

Both lines describe phonon assisted tunneling from $\mathbf{k}, b$ to $\mathbf{k}+\mathbf{K}, \bar{b}$. However, in the first line first the tunneling and next the phonon scattering takes place, whereas in the second line, first the phonon scattering and next the tunneling takes place. Considering the intermediate states$\mathbf{k}, \bar{b}$ and $\mathbf{k}+\mathbf{K}, b$ being much larger in energy, in both lines, all contributions have the same sign and add up. The relative sign between both lines $(-)$ is compensated by the opposite signs of the appearing u and s functions. To further evaluate the phonon assisted tunnel Hamiltonian, we carry out the summation over the layer

$$\begin{aligned} H = &\frac{1}{2}\sum_{\mathbf{k}\mathbf{K}\lambda G\xi} \lambda_{\mathbf{k}+\mathbf{K}}^{\dagger W}\lambda_{\mathbf{k}}^{G}\left(\underbrace{t^{\lambda GW} g_{\mathbf{K}}^{\lambda W\xi}(\alpha_{\mathbf{k}}^{\lambda GW} + \gamma_{\mathbf{k},\mathbf{K}}^{\lambda W\xi})}_{s_{\mathbf{k},\mathbf{K}}^{\lambda GW\xi}} b_{\mathbf{K}}^{W\xi} + \underbrace{t^{\lambda GW} g_{\mathbf{K}}^{\lambda W\xi}(\alpha_{\mathbf{k}}^{\lambda GW} + \beta_{\mathbf{k},\mathbf{K}}^{\lambda W\xi})}_{\tilde{s}_{\mathbf{k},\mathbf{K}}^{\lambda GW\xi}} b_{-\mathbf{K}}^{\dagger W\xi}\right) \\ &- \frac{1}{2}\sum_{\mathbf{k}\mathbf{K}\lambda W\xi} \lambda_{\mathbf{k}+\mathbf{K}}^{\dagger G}\lambda_{\mathbf{k}}^{W}\left(\underbrace{t^{\lambda GW} g_{\mathbf{K}}^{\lambda W\xi}(\alpha_{\mathbf{k}+\mathbf{K}}^{\lambda WG} + \gamma_{\mathbf{k},\mathbf{K}}^{\lambda W\xi})}_{u_{\mathbf{k},\mathbf{K}}^{\lambda WG\xi}} b_{\mathbf{K}}^{W\xi} + \underbrace{t^{\lambda GW} g_{\mathbf{K}}^{\lambda W\xi}(\alpha_{\mathbf{k}+\mathbf{K}}^{\lambda WG} + \beta_{\mathbf{k},\mathbf{K}\xi}^{\lambda W})}_{\tilde{u}_{\mathbf{k},\mathbf{K}}^{\lambda WG\xi}} b_{-\mathbf{K}}^{\dagger W\xi}\right) \\ &+ \frac{1}{2}\sum_{\mathbf{k}\mathbf{K}\lambda\xi} \lambda_{\mathbf{k}+\mathbf{K}}^{\dagger G}\lambda_{\mathbf{k}}^{W}\left(\underbrace{t^{\lambda WG} g_{\mathbf{K}}^{\lambda G\xi}(\alpha_{\mathbf{k}}^{\lambda WG} + \gamma_{\mathbf{k},\mathbf{K}}^{\lambda G\xi})}_{s_{\mathbf{k},\mathbf{K}}^{\lambda WG\xi}} b_{\mathbf{K}}^{G\xi} + \underbrace{t^{\lambda WG} g_{\mathbf{K}}^{\lambda G\xi}(\alpha_{\mathbf{k}}^{\lambda WG} + \beta_{\mathbf{k},\mathbf{K}}^{\lambda G\xi})}_{\tilde{s}_{\mathbf{k},\mathbf{K}}^{\lambda WG\xi}} b_{-\mathbf{K}}^{\dagger G\xi}\right) \\ &- \frac{1}{2}\sum_{\mathbf{k}\mathbf{K}\lambda\xi} \lambda_{\mathbf{k}+\mathbf{K}}^{\dagger W}\lambda_{\mathbf{k}}^{G}\left(\underbrace{t^{\lambda WG} g_{\mathbf{K}}^{\lambda G\xi}(\alpha_{\mathbf{k}+\mathbf{K}}^{\lambda GW} + \gamma_{\mathbf{k},\mathbf{K}}^{\lambda G\xi})}_{u_{\mathbf{k},\mathbf{K}}^{\lambda GW\xi}} b_{\mathbf{K}}^{G\xi} + \underbrace{t^{\lambda WG} g_{\mathbf{K}}^{\lambda G\xi}(\alpha_{\mathbf{k}+\mathbf{K}}^{\lambda GW} + \beta_{\mathbf{k},\mathbf{K}}^{\lambda G\xi})}_{\tilde{u}_{\mathbf{k},\mathbf{K}}^{\lambda GW\xi}} b_{-\mathbf{K}}^{\dagger G\xi}\right) \end{aligned} \tag{83}$$

Here, the first two lines involve phonons in WSe_2 whereas the last two lines involve phonons

in graphene. The $-$ signs in front of line two and line four are compensated by the opposite signs of the s and u functions. To investigate the different contributions to the tunnel-phonon coupling in more detail, we evaluate the functions s and u in the different terms: (a) we fix the momenta in WSe_2 to the vicinity of the K point. (b) While all scattering processes conserve the energy, the approximation (a) settles the energy and momentum range of involved carriers. (c) In the next step, we approximate the prefactors $\alpha_{\mathbf{k}}^{\lambda ij}$ by their values in the region of interest, where we find $\alpha_{\mathbf{k}\approx K_W}^{cGW} = \frac{1}{1\text{eV}}$ and $\alpha_{\mathbf{k}\approx K_G}^{cWG} = \frac{1}{250\text{meV}}$, which are read out from the DFT calculation in the main manuscript. To evaluate the prefactors $\beta_{\mathbf{k},\mathbf{K}}^{\lambda i\xi}$ and $\gamma_{\mathbf{k},\mathbf{K}}^{\lambda i\xi}$ we first realize, that that ΔE_{K_G} appears whenever WSe_2 phonons are involved, and ΔE_{K_W} appears whenever graphene phonons are involved. ΔE_{K_G} $\approx$250 meV is large in comparison to typical phonon energies of 30 meV in WSe_2 and ΔE_{K_W} $\approx$1 eV is large in comparison to typical phonon energies of 200 meV in graphene. Consequently we ignore the appearing phonon energies in $\beta_{\mathbf{k},\mathbf{K}}^{\lambda i\xi}$ and $\gamma_{\mathbf{k},\mathbf{K}}^{\lambda i\xi}$. As a result, the Hamiltonian simplifies to

$$\begin{aligned} H = &\sum_{\mathbf{kK}\lambda,\xi} \underbrace{\frac{t^{\lambda GW} g_{\mathbf{K}}^{\lambda W\xi}}{\epsilon_{K_{gr}}^{W} - \epsilon_{K_{gr}}^{G}}}_{h_{\mathbf{K}}^{W\xi}\approx const.} \lambda_{\mathbf{k}+\mathbf{K}}^{\dagger W} \lambda_{\mathbf{k}}^{G} \left(b_{\mathbf{K}}^{W\xi} + b_{-\mathbf{K}}^{\dagger W\xi} \right) + h.c. \\ + &\sum_{\mathbf{kK}\lambda\xi} \underbrace{\frac{t^{\lambda WG} g_{\mathbf{K}}^{\lambda G\xi}}{\epsilon_{K_W}^{G} - \epsilon_{K_W}^{W}}}_{h_{\mathbf{K}}^{G}\approx const.} \lambda_{\mathbf{k}+\mathbf{K}}^{\dagger W} \lambda_{\mathbf{k}}^{G} \left(b_{\mathbf{K}}^{G\xi} + b_{-\mathbf{K}}^{\dagger G\xi} \right) + h.c.\,. \end{aligned} \tag{84}$$

For a carrier in graphene, the relaxation rate to WSe_2 via phonon-assisted tunneling is given

as

$$\Gamma^G_{\mathbf{k}} = 2\pi \sum_{\pm,\mathbf{K},\xi,i\in\{W,G\}} |h^{i\xi}_{\mathbf{K}}|^2 \left(\frac{1}{2} \pm \frac{1}{2} + n^{i\xi}_{\mathbf{K}}\right) \delta(\epsilon^G_{\mathbf{k}} - \epsilon^W_{\mathbf{k}+\mathbf{K}} \mp \hbar\omega^{i\xi}_{\mathbf{K}}) \tag{85}$$

Assuming $h^{i\xi}_{\mathbf{K}} \approx h^{i\xi}$ and $\hbar\omega^{i\xi}_{\mathbf{K}} \approx \hbar\omega^{i\xi}$, we obtain

$$\Gamma^G_{\mathbf{k}} = A \sum_{i\in\{W,G\},\xi,\pm} \frac{m^W}{\hbar^2} |h^i|^2 \left(\frac{1}{2} \pm \frac{1}{2} + n^{i\xi}\right) 1_{\epsilon^G_{\mathbf{k}} \mp \hbar\omega^{i\xi} - \epsilon^W_{\mathbf{0}} > 0}, \tag{86}$$

which is constant for graphene electrons which have at least the energy of the conduction band plus the phonon energy in graphene. This reflects the constant density of states in WSe_2. The area A cancels with the area in the phonon coupling element which is contained in h^i.

In contrast for carriers initially located in WSe_2, we get

$$\Gamma^W_{\mathbf{k}} = 2\pi \sum_{\pm,\mathbf{K},\xi,i\in\{W,G\}} |h^{i\xi}_{\mathbf{K}}|^2 \left(\frac{1}{2} \pm \frac{1}{2} + n^{i\xi}_{\mathbf{K}}\right) \delta(\epsilon^W_{\mathbf{k}} - \epsilon^G_{\mathbf{k}+\mathbf{K}} \mp \hbar\omega^{i\xi}_{\mathbf{K}}). \tag{87}$$

With similar approximation as above, we end up at

$$\Gamma^W_{\mathbf{k}} = A \sum_{i\in\{W,G\},\xi,\pm} \frac{\epsilon_{\mathbf{k}} \mp \hbar\omega^{i\xi}}{\hbar^2 v_F^2} |h^i|^2 \left(\frac{1}{2} \pm \frac{1}{2} + n^{i\xi}\right), \tag{88}$$

To calculate the relaxation rates, we assume a potential barrier of E_B =5 eV corresponding to the energy of the WSe_2 conduction band w.r.t. to the vacuum level. Then we approximate the tunneling element as $t = \chi E_B$, with χ being the wavefunction overlap[29]. For graphene, we include two optical phonon branches with energies of $\hbar\omega$ =200 meV and the coupling strength of g =200 meV[42]. For WSe_2, we include two optical phonon branches with energies of $\hbar\omega$ =30 meV

and the coupling strength of $g = 10\,\text{meV}$[43]. Supplementary Fig. 15**a** illustrates the approximate relaxation rates of electrons from graphene to WSe_2 (Γ^G) and WSe_2 to graphene (Γ^W) as a function of the overlap of the wavefunctions between WSe_2 and graphene. We find for both a quadratic increase as a function of the overlap, since the latter enters quadratic in both relaxation rates. The difference between both relaxation rates arise from different final densities of states of the carrier relaxation. In the experiment, a delayed rise of the WSe_2 signal w.r.t. to the graphene signal of about 50 fs (13 meV) was found, which indicates an overlap of 4.0%. The order of magnitude of this value appears reasonable, since the overlap between two neighboring graphene atoms is 7% as an example[35].

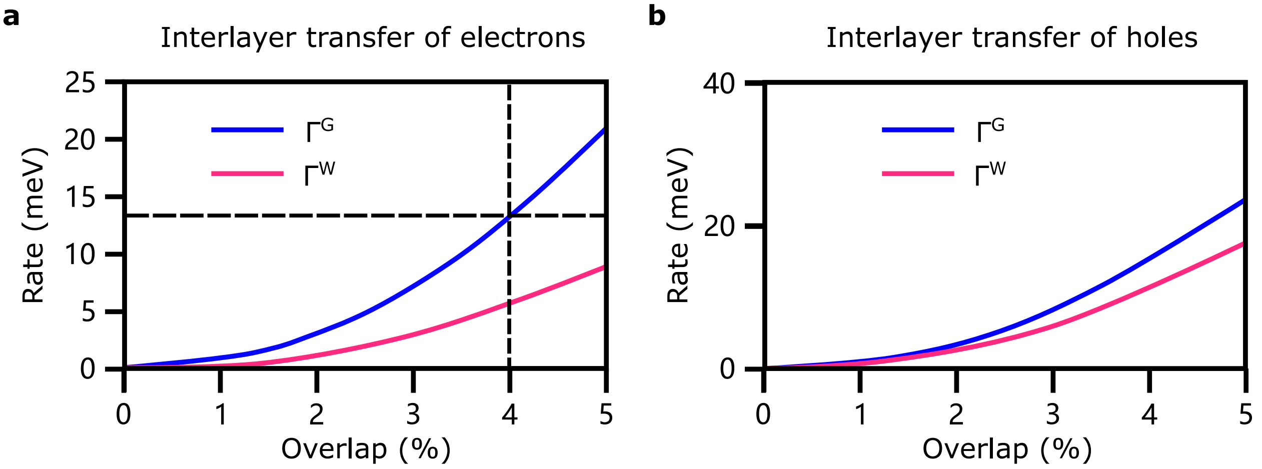

Supplementary Fig. 15: Tunnel transfer for electrons and holes. a, The ICT-induced relaxation rate of electrons in graphene towards WSe_2 (blue) as a function of the electronic wavefunction overlap between the involved conduction bands. The relaxation rate of electrons in WSe_2 towards graphene (pink). The dashed lines indicate the overlapping values which can be expected from the experiment results. **b**, The relaxation rate of

holes in graphene, which have larger energies compared to the valence band maximum in WSe_2 (blue) and the relaxation rate of holes in WSe_2 (pink).

To evaluate the phonon-assisted tunneling rates for holes, we assume similar phonon coupling elements in conduction and valence band in WSe_2[43] and graphene but account for the different dispersion of the valence band[44]. Supplementary Fig. 15**b** illustrates the tunneling rates of holes. We find qualitatively similar tunneling rates as for electrons. However, the tunneling of holes from WSe_2 to graphene is stronger compared to the electrons. This arises from the larger density of states of graphene for the involved final states. The reason for this, is that the Fermi energy of the system is closer to the conduction band minimum compared to the valence band maximum, cp. Fig. 2**a** in the manuscript.

Table 1: Parameters used in the computation. * exemplary value at $z = 0$ determined numerically by evaluating the Wannier equation for WSe_2 on a SiC substrate. [27,45]. ** taken as double distance between the chalcogen atoms. The Fermi velocity taken the experimental result.

Param.		Param.		Ref.
$\hbar$	0.658 eV fs	d_G	0.25 e nm	[46]
e	1 e	v_F	1.8 nm fs^{-1}	exp
ϵ_0	5.5·10^{-2} e^{2}eV^{-1}nm^{-1}	d_{WSe_2}	0.32 e nm	[27]
k_B	8.6·10^{-5} eV K^{-1}	$\lvert\varphi_{WSe_2}(\mathbf{r}=0)\rvert$	0.36 nm^{-1}	*
ϵ_{SiC}	9.6	$E^{1s}_{WSe_2}$	1.7 eV	[47]
		M_{WSe_2}	3.7 eVfs^{2}nm^{-2}	[20]
ϵ_G	6[48]	ϵ_{WSe_2}	13.36	
a_G	0.33 nm [49]	a_{WSe_2}	0.67 nm	** [50]

References

1. Zhao, W. *et al.* Lattice dynamics in mono-and few-layer sheets of WS_2 and WSe_2. *Nanoscale* **5**, 9677–9683 (2013).

2. Terrones, H. *et al.* New first order Raman-active modes in few layered transition metal dichalcogenides. *Sci. Rep.* **4**, 4215 (2014).

3. Tonndorf, P. *et al.* Photoluminescence emission and Raman response of monolayer MoS_2, $MoSe_2$, and WSe_2. *Opt. Express* **21**, 4908–4916 (2013).

4. He, K. *et al.* Tightly bound excitons in monolayer WSe_2. *Phys. Rev. Lett.* **113**, 026803 (2014).

5. Froehlicher, G., Lorchat, E. & Berciaud, S. Charge versus energy transfer in atomically thin graphene-transition metal dichalcogenide van der Waals heterostructures. *Phys. Rev. X* **8**, 011007 (2018).

6. Nakamura, H. *et al.* Spin splitting and strain in epitaxial monolayer WSe_2 on graphene. *Phys. Rev. B* **101**, 165103 (2020).

7. Maklar, J. *et al.* A quantitative comparison of time-of-flight momentum microscopes and hemispherical analyzers for time-resolved ARPES experiments *Rev. Sci. Instrum.* **91**, 123112 (2020).

8. Gierz, I., Henk, J., Höchst, H., Ast, C. R. & Kern, K. Illuminating the dark corridor in graphene: Polarization dependence of angle-resolved photoemission spectroscopy on graphene. *Phys. Rev. B* **83**, 121408 (2011).

9. Hwang, C. *et al.* Fermi velocity engineering in graphene by substrate modification. *Sci. Rep.* **2**, 590 (2012).

10. Siegel, D. A. *et al.* Many-body interactions in quasi-freestanding graphene. *PNAS* **108**, 11365–11369 (2011).

11. Yuan, L., Wang, T., Zhu, T., Zhou, M. & Huang, L. Exciton dynamics, transport, and annihilation in atomically thin two-dimensional semiconductors. *J. Phys. Chem. Lett.* **8**, 3371–3379 (2017).

12. Wang, H., Zhang, C. & Rana, F. Ultrafast dynamics of defect-assisted electron–hole recombination in monolayer MoS_2. *Nano Lett.* **15**, 339–345 (2015).

13. Dendzik, M. *et al.* Observation of an excitonic Mott transition through ultrafast core-*cum*-conduction photoemission spectroscopy. *Phys. Rev. Lett.* **125**, 096401 (2020).

14. Liu, F., Ziffer, M. E., Hansen, K. R., Wang, J. & Zhu, X. Direct determination of band-gap renormalization in the photoexcited monolayer MoS_2. *Phys. Rev. Lett.* **122**, 246803 (2019).

15. Ruppert, C., Chernikov, A., Hill, H. M., Rigosi, A. F. & Heinz, T. F. The role of electronic and phononic excitation in the optical response of monolayer WS_2 after ultrafast excitation. *Nano Lett.* **17**, 644–651 (2017).

16. Tomadin, A., Brida, D., Cerullo, G., Ferrari, A. C. & Polini, M. Nonequilibrium dynamics of photoexcited electrons in graphene: Collinear scattering, auger processes, and the impact of screening. *Phys. Rev. B* **88**, 035430 (2013).

17. Liang, Y. & Yang, L. Carrier plasmon induced nonlinear band gap renormalization in two-dimensional semiconductors. *Phys. Rev. Lett.* **114**, 063001 (2015).

18. Stimper, V., Bauer, S., Ernstorfer, R., Schölkopf, B. & Xian, R. P. Multidimensional contrast limited adaptive histogram equalization. *IEEE Access* **7**, 165437–165447 (2019).

19. Xian, R. P. *et al.* A machine learning route between band mapping and band structure. *arXiv:2005.10210* (2020).

20. Kormányos, A. *et al.* k· p theory for two-dimensional transition metal dichalcogenide semiconductors. *2D Mater.* **2**, 022001 (2015).

21. Selig, M. *et al.* Dark and bright exciton formation, thermalization, and photoluminescence in monolayer transition metal dichalcogenides. *2D Mater.* **5**, 035017 (2018).

22. Yeh, P.-C. *et al.* Layer-dependent electronic structure of an atomically heavy two-dimensional dichalcogenide. *Phys. Rev. B* **91**, 041407 (2015).

23. Zhang, Y. *et al.* Electronic structure, surface doping, and optical response in epitaxial wse2 thin films. *Nano Lett.* **16**, 2485–2491 (2016).

24. Harb, M. *et al.* Carrier relaxation and lattice heating dynamics in silicon revealed by femtosecond electron diffraction. *J. Phys. Chem. B* **110**, 25308–25313 (2006).

25. Hong, J., Senga, R., Pichler, T. & Suenaga, K. Probing exciton dispersions of freestanding monolayer WSe_2 by momentum-resolved electron energy-loss spectroscopy. *Phys. Rev. Lett.* **124**, 087401 (2020).

26. Selig, M. *et al.* Ultrafast dynamics in monolayer transition metal dichalcogenides: interplay of dark excitons, phonons, and intervalley exchange. *Phys. Rev. Res.* **1**, 022007 (2019).

27. Selig, M. *et al.* Excitonic linewidth and coherence lifetime in monolayer transition metal dichalcogenides. *Nat. Commun.* **7**, 13279 (2016).

28. Dong, S. *et al.* Direct measurement of key exciton properties: Energy, dynamics, and spatial distribution of the wave function. *Nat. Sci.* **1**,e10010 (2021).

29. Ovesen, S. *et al.* Interlayer exciton dynamics in van der Waals heterostructures. *Commun. Phys.* **2**, 1–8 (2019).

30. Katsch, F., Selig, M. & Knorr, A. Theory of coherent pump–probe spectroscopy in monolayer transition metal dichalcogenides. *2D Mater.* **7**, 015021 (2019).

31. Selig, M., Malic, E., Ahn, K. J., Koch, N. & Knorr, A. Theory of optically induced Förster coupling in van der Waals coupled heterostructures. *Phys. Rev. B* **99**, 035420 (2019).

32. Rana, F. Electron-hole generation and recombination rates for coulomb scattering in graphene. *Phys. Rev. B* **76**, 155431 (2007).

33. Wendler, F., Knorr, A. & Malic, E. Carrier multiplication in graphene under landau quantization. *Nat. Commun.* **5**, 3703 (2014).

34. Dexter, D. L. A theory of sensitized luminescence in solids. *J. Chem. Phys.* **21**, 836–850 (1953).

35. Reich, S., Maultzsch, J., Thomsen, C. & Ordejon, P. Tight-binding description of graphene. *Phys. Rev. B* **66**, 035412 (2002).

36. Chernikov, A. *et al.* Exciton binding energy and nonhydrogenic Rydberg series in monolayer WS_2. *Phys. Rev. Lett.* **113**, 076802 (2014).

37. Wu, F., Qu, F. & Macdonald, A. H. Exciton band structure of monolayer MoS_2. *Phys. Rev. B* **91**, 075310 (2015).

38. Robert, C. *et al.* Optical spectroscopy of excited exciton states in MoS_2 monolayers in van der Waals heterostructures. *Phys. Rev. Mater.* **2**, 011001 (2018).

39. Rytova, N. S. Screened potential of a point charge in a thin film. *arXiv:1806.00976* (2018).

40. Keldysh, L. Coulomb interaction in thin semiconductor and semimetal films. *J. Exp. Theor. Phys.* **29**, 658 (1979).

41. Cudazzo, P., Tokatly, I. V. & Rubio, A. Dielectric screening in two-dimensional insulators: Implications for excitonic and impurity states in graphene. *Phys. Rev. B* **84**, 085406 (2011).

42. Piscanec, S., Lazzeri, M., Mauri, F., Ferrari, A. & Robertson, J. Kohn anomalies and electron-phonon interactions in graphite. *Phys. Rev. Lett.* **93**, 185503 (2004).

43. Jin, Z., Li, X., Mullen, J. T. & Kim, K. W. Intrinsic transport properties of electrons and holes in monolayer transition-metal dichalcogenides. *Phys. Rev. B* **90**, 045422 (2014).

44. Kormányos, A., Zólyomi, V., Drummond, N. D. & Burkard, G. Spin-orbit coupling, quantum dots, and qubits in monolayer transition metal dichalcogenides. *Phys. Rev. X* **4**, 011034 (2014).

45. Berghäuser, G. & Malic, E. Analytical approach to excitonic properties of MoS_2. *Phys. Rev. B* **89**, 125309 (2014).

46. Malic, E., Winzer, T., Bobkin, E. & Knorr, A. Microscopic theory of absorption and ultrafast many-particle kinetics in graphene. *Phys. Rev. B* **84**, 205406 (2011).

47. Christiansen, D. *et al.* Phonon sidebands in monolayer transition metal dichalcogenides. *Phys. Rev. Lett.* **119**, 187402 (2017).

48. Bessler, R., Duerig, U. & Koren, E. The dielectric constant of a bilayer graphene interface. *Nanoscale Adv.* **1**, 1702–1706 (2019).

49. Ye, S. *et al.* Thickness-dependent strain effect on the deformation of the graphene-encapsulated Au nanoparticles. *J. Nanomater.* **2014** (2014).

50. Berkelbach, T. C., Hybertsen, M. S. & Reichman, D. R. Theory of neutral and charged excitons in monolayer transition metal dichalcogenides. *Phys. Rev. B* **88**, 045318 (2013).
