# Supplementary material for: Observation of ultrafast interfacial Meitner-Auger energy transfer in a Van der Waals heterostructure
Source: Nat Commun. 2023 Aug 19;14:5057. doi: 10.1038/s41467-023-40815-8 (PMC10439896; doi:10.1038/s41467-023-40815-8)
Supplement: Supplementary file 2 — Peer Review File [file 41467_2023_40815_MOESM2_ESM.pdf]

nature portfolio

Peer Review File

Observation of ultrafast interfacial Meitner-Auger energy transfer in a van der Waals heterostructure

Open Access This file is licensed under a Creative Commons Attribution 4.0 International License, which permits use, sharing, adaptation, distribution and reproduction in any medium or format, as long as you give appropriate credit to the original author(s) and the source, provide a link to the Creative Commons license, and indicate if changes were made. In the cases where the authors are anonymous, such as is the case for the reports of anonymous peer reviewers, author attribution should be to 'Anonymous Referee' followed by a clear attribution to the source work. The images or other third party material in this file are included in the article's Creative Commons license, unless indicated otherwise in a credit line to the material. If material is not included in the article's Creative Commons license and your intended use is not permitted by statutory regulation or exceeds the permitted use, you will need to obtain permission directly from the copyright holder. To view a copy of this license, visit http://creativecommons.org/licenses/by/4.0/.

Editorial Note: This manuscript has been previously reviewed at another journal that is not operating a transparent peer review scheme. This document only contains reviewer comments and rebuttal letters for versions considered at *Nature Communications*.

Reviewers' Comments:

Reviewer #1:
Remarks to the Author:
The manuscript has been heavily revised in the last iterations, and I believe the ms can be published in Nature communication with the current form without any further revision.

Revision and resubmission of manuscript NCOMMS-23-28105-T

Reviewer #1 (Remarks to the Author):

The manuscript has been heavily revised in the last iterations, and I believe the ms can be published in Nature communication with the current form without any further revision.

We thank the Referee for supporting the publication of our manuscript in Nature Communications.
